# Coxsackievirus B escapes antiviral CD8^+^ T cells but triggers robust CD4^+^ memory responses

**DOI:** 10.1126/sciadv.aef5067

**Published:** 2026-06-19

**Authors:** Orlando Burgos-Morales, Federica Vecchio, Fatoumata Samassa, Margot Petit, Zhicheng Zhou, Barbara Brandao, Alexia Carré, Annalisa Nicastri, Valeriia Dotsenko, Chloe Shepherd, Keijo Viiri, Robert Parker, Amelia K. Linnemann, Sylvaine You, Malin Flodstrom-Tullberg, Nicola Ternette, Roberto Mallone

**Affiliations:** ^1^ Université Paris Cité, Institut Cochin, CNRS, INSERM, Paris, France.; ^2^ School of Life Sciences, University of Dundee, Dundee, Scotland, UK.; ^3^ Faculty of Medicine and Health Technology, Celiac Disease Research Center, Tampere University and Tampere University Hospital, Tampere, Finland.; ^4^ Faculty of Biochemistry and Molecular Medicine Oulu, University of Oulu, Oulu, Finland.; ^5^ Centre for Immuno-Oncology, University of Oxford, Oxford, UK.; ^6^ Center for Diabetes and Metabolic Diseases, Indiana University School of Medicine, Indianapolis, IN, USA.; ^7^ Indiana Biosciences Research Institute, Indianapolis, IN, USA.; ^8^ Karolinska University Hospital Huddinge, Department of Medicine Huddinge, Center for Infectious Medicine, Karolinska Institutet, Stockholm, Sweden.; ^9^ Assistance Publique Hôpitaux de Paris, Cochin Hospital, Service de Diabétologie et Immunologie Clinique, Paris, France.

## Abstract

Coxsackieviruses B (CVBs) are plausible triggers of the pancreatic islet autoimmunity leading to type 1 diabetes. Islet autoantibody seroconversion correlates with persistent CVB infections in the gut and pancreas, suggesting defective antiviral control and the need to define immune mechanisms at the intestinal entry site. We investigated how CVB3 modulates antigen presentation, the viral immunopeptidome of enterocytes and antigen-presenting cells, and downstream T cell immunity. CVB3-infected enterocytes evaded immune recognition by down-regulating human leukocyte antigen (HLA) class I and viral peptide presentation, impairing CD8^+^ T cell responses in vitro. In CVB-seropositive individuals, circulating CVB-reactive CD8^+^ T cells were stalled in naïve-like and exhausted effector/memory states. In contrast, CVB3 induced HLA class II up-regulation, promoting robust CD4^+^ T cell activation. Circulating CVB3-reactive CD4^+^ T cells fully differentiated into polyfunctional T helper memory. These findings indicate that CVB3 antiviral control is predominantly CD4^+^ T cell mediated and provide a rationale for mucosal vaccination strategies and immune monitoring tools to follow infection or vaccination.

## Teaser

Coxsackievirus evades CD8^+^ T cell immunity in the gut, leaving CD4^+^ T cells as the main line of antiviral defense.

## INTRODUCTION

Type 1 diabetes (T1D) is an autoimmune disease of unknown etiology. Its increasing incidence, including in individuals harboring neutral or protective human leukocyte antigen class II (HLA-II) haplotypes, suggests that environmental triggers may play a primary role in disease onset (*1*). These triggers likely have their greatest impact early in life, as a large proportion of children who later develop T1D exhibit islet autoantibody (aAb) seroconversion before the age of 2 (*2*).

Coxsackieviruses B (CVBs) are small, nonenveloped, positive-sense single-stranded RNA viruses belonging to the *Enterovirus* genus within the Picornaviridae family. Their genome encodes a single polyprotein that is cleaved into four structural capsid proteins [viral protein 1 (VP1) to VP4; the P1 region forming the mature virion] and seven nonstructural proteins involved in viral replication (P2 and P3 regions). Histopathological and epidemiological studies point to CVBs as plausible triggers of the autoimmune T cell attack against pancreatic islet β cells (*3*). The histopathological colocalization of the enteroviral VP1 and double-stranded RNA (dsRNA) with HLA class I (HLA-I) hyperexpression (a recognized hallmark of disease) in the islets of T1D donors (*4*–*6*) suggests that low-grade, persistent CVB infections may trigger the autoimmune process (*7*). In line with these observations, stool metagenome sequencing on prospective cohorts of genetically at-risk children demonstrated a significant correlation between prolonged CVB infections (characterized by the persistent shedding of the same CVB serotype in sequential stool samples) and the aAb seroconversion marking the initiation of islet autoimmunity (*8*). These persistent CVB infections (*7*), possibly facilitated by inefficient antiviral immune responses in predisposed individuals (*3*, *9*), may contribute to T1D autoimmunity.

While a causal link between CVBs and the onset of T1D autoimmunity remains elusive, the positive results of recent clinical trials may provide this missing link and novel intervention and prevention strategies by enhancing antiviral responses. The antiviral agents pleconaril and ribavirin provided some preservation of residual insulin secretion in children and adolescents with new-onset clinical (stage 3) T1D (*10*). In addition, T1D primary prevention trials are being considered to explore the protective effect of a multivalent inactivated CVB vaccine (*11*–*13*). These trials aim to directly assess whether preventing CVB infection by vaccination can offer protection against islet autoimmunity and subsequent T1D development, potentially providing proof for the viral etiology of the disease and a primary prevention option not available to date.

Several knowledge gaps, however, exist about the natural immune response against CVB (*3*). One serological study suggests that children who later develop anti-insulin aAbs at an early age do not harbor anti-CVB neutralizing antibodies (Abs) (*14*). This could potentially make them more susceptible to infections with higher viral loads, increasing the likelihood of high-load viremia and CVB spreading to the pancreas (*3*). On the same lines, we have previously documented that CVB infects and destroys β cells but elicits limited antiviral CD8^+^ T cell responses (*9*). Overall, these observations provide a strong rationale for CVB vaccination.

Because CVB enters the body mainly through gut epithelia before infecting β cells, both tissue types are relevant to understand the mechanisms by which CVB may trigger T1D. Available studies also show that persistent infections in mucosal tissues are strongly associated with T1D (*15*, *16*), suggesting that impaired antiviral immunity at the entry site may contribute to disease onset. However, the nature of T cell responses, and how CVB modulates antigen presentation in the intestinal mucosa remain poorly understood. Here, we addressed this gap by focusing on antigen presentation as a critical determinant of T cell–mediated immunity, using two complementary models of infection: intestinal epithelial cells and professional antigen-presenting cells (APCs). Through an integrated proteomics and immunopeptidomics strategy, we uncovered viral modulation of antigen processing pathways in enterocytes, characterized by reduced HLA-I but increased HLA-II expression, and a specific repertoire of CVB peptides presented. These experiments were complemented with a macrophage model to map the HLA-II–restricted viral peptides presented upon uptake of cell debris from CVB-infected cells. These datasets enabled us to define previously unknown HLA-A*02:01– and HLA-DRB1*04:01–restricted CVB epitopes and assess their immunogenicity. In line with the differential effect on HLA-I and HLA-II modulation, our results reveal selective impairment of CD8^+^ T cell responses alongside robust, polyfunctional CD4^+^ T cell responses, highlighting CVB-driven immune evasion strategies that favor CD4^+^ T cells.

## RESULTS

### CVB infection of CaCo2 enterocytes reveals immune escape mechanisms and hijacking of host cell machinery

**Fig. 1.**
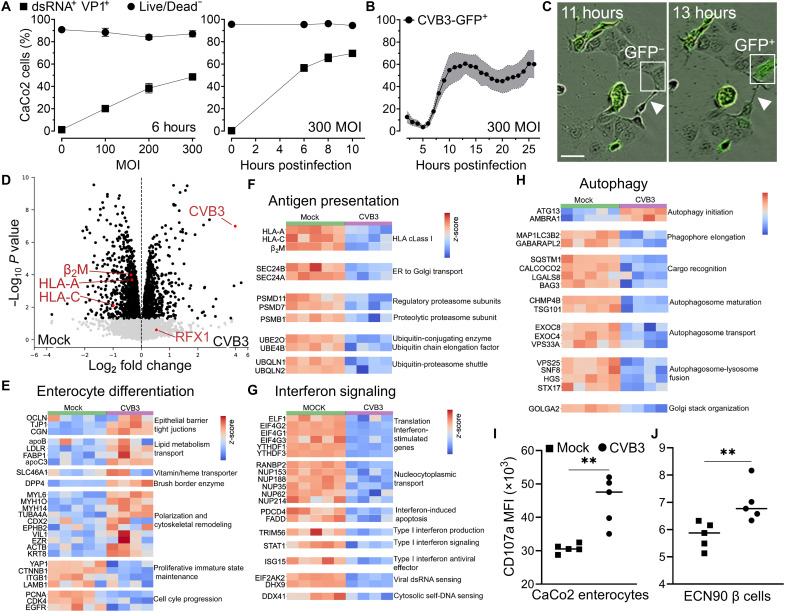
CVB3 immune escape mechanisms in infected CaCo2 enterocytes. (**A**) Setup of the in vitro infection protocol by flow cytometry: optimization of the 300 MOI CVB3 load (left; three replicates) and 10-hour infection time course (right; four replicates) to achieve maximal infection rates (percent dsRNA^+^VP1^+^ cells) without cell death (percent Live/Dead^−^ cells). (**B**) Infection time course using a fluorescent CVB3-GFP strain (300 MOI), measured by live-cell imaging for 25 hours (eight replicates). Data in (A) and (B) represent mean ± SD from a representative experiment performed in triplicate. (**C**) Phase contrast images overlaid with GFP fluorescence of CVB3-GFP–infected CaCo2 enterocytes at 11 and 13 hours. Arrows point to filopodia. Scale bar, 50 μm. (**D**) Proteomics analysis of CVB3-infected (*n* = 4) versus mock-infected (*n* = 5) CaCo2 enterocytes (300 MOI; 10 hours). Volcano plot of differentially expressed proteins (black dots) with a significance threshold of *P* < 0.05. RFX1, regulatory factor X1. (**E** to **H**) Functional categories of proteins differentially expressed: proteins related to enterocyte differentiation (E), antigen processing and presentation (F), interferon (IFN) signaling (G), and autophagy (H). n.s., not significant. (**I** and **J**) Surface expression [median fluorescence intensity (MFI)] of the lysosomal marker CD107a (LAMP1) in CVB3- versus mock-infected CaCo2 enterocytes [(I); 100 MOI, 20 hours] and ECN90 β cells [(J); 50 MOI, 16 hours]. Bars represent median values of five replicates from a representative experiment performed in duplicate. **P = 0.008 by Mann-Whitney *U* test.

To investigate the early intestinal mucosal phase of CVB infection, we established an in vitro model using CaCo2 enterocytes, which express both HLA-I (A*02:01, A2 from hereon; B*15:01; and C*04:01) and HLA-II molecules (DRB1*04:04, DRB4*04:01, DQA1*03:01, DQB1*03:02, DPA1*01:03, and DPB1*04:01). We first titrated multiplicities of infection (MOI) using a prototypic CVB3 strain and determined the percentage of infected CaCo2 enterocytes by flow cytometry measurement of the intracellular capsid VP1 and viral replication intermediate dsRNA at 6 hours postinfection (hpi) (*9*). Infection rates increased with MOI, reaching a maximum of ∼49% infected cells at 300 MOI while preserving cell viability (Fig. 1A, left, and representative staining profiles in fig. S1). We then determined whether the proportion of infected cells could be further increased by extending the infection to 8 and 10 hpi. The percentage of infected VP1^+^dsRNA^+^ cells reached a plateau at ∼70% after 10 hpi, again with minimal impact on cell viability (Fig. 1A, right). To validate this infection protocol, we used a CVB3–green fluorescent protein (GFP) strain to track infection kinetics in CaCo2 cells by live-cell fluorescence imaging (*9*). Infection (here identified as GFP signal) exhibited an initial lag phase up until 5 hpi, followed by an exponential increase in GFP^+^ cells that plateaued at ∼10 hpi (Fig. 1B), in line with flow cytometry results. Live-cell imaging further revealed morphological changes in infected enterocytes similar to those we previously observed in pancreatic β cells (*9*): Cell-to-cell contact through filopodia-like projections allowed cells to transition from noninfected (GFP^−^) to infected (GFP^+^) state (Fig. 1C), suggesting that CVB3-induced cytoskeletal remodeling contributes to viral transfer.

To understand how CVB3 modulates host cellular pathways, we performed quantitative proteomics on CVB3- versus mock-infected CaCo2 enterocytes. Among the 6944 proteins detected, 1125 (16.2%) were differentially expressed (Fig. 1D). As detailed below, CVB3 infection induced an extensive proteome remodeling, consistent with a coordinated viral strategy to skew enterocyte development and suppress immune recognition.

First, CVB3 infection shifted enterocyte identity toward a more differentiated and polarized phenotype (Fig. 1E). Tight junction proteins (OCLN, TJP1, and CGN), cytoskeletal and apical polarity markers (MYH10, MYL6, VIL1, CDX2, TUBA4A, KRT8, ACTB, and EZR), and the brush border enzyme DPP4 were up-regulated. Lipid and vitamin transport proteins (apoB, LDLR, FABP, apoC3, SLC46A1) were also increased, suggesting maintenance of absorptive functions despite infection. In contrast, CVB3 infection reduced the expression of key regulators of proliferation and tissue renewal, including YAP1, CTNNB1 (β-catenin), ITGB1 and LAMB1, along with cell cycle effectors PCNA, CDK4, and EGFR. These proteins are involved in maintaining enterocyte progenitor pools (*17*), promoting regenerative signaling (*18*), and driving G_1_-S cell cycle progression (*19*). This may reflect a viral strategy to arrest the host cell cycle, particularly at the G_1_ phase, as previously reported in enteroviral infections (*20*). Collectively, these changes maintain a polarized mature phenotype in enterocytes, altering epithelial barrier integrity due to impaired cell replacement.

Second, multiple components of the antigen processing and presentation pathways were down-regulated (Fig. 1F), including HLA-I (HLA-A and HLA-C but not HLA-B) and β_2_-microglobulin (β2M), thus impairing surface expression of peptide (p)HLA-I complexes essential for CD8^+^ T cell recognition. This down-regulation extended to the proteasome, with reduced expression of regulatory (PSMD7 and PSMD11) and catalytic (PSMB1) subunits needed for antigenic peptide processing. Endoplasmic reticulum (ER)–to–Golgi transport mediators SEC24A and SEC24B, which facilitate HLA-I trafficking, were also reduced. Furthermore, proteins involved in the ubiquitin-proteasome system, specifically the conjugation enzyme UBE2O, elongation factor UBE4B, and shuttling factors UBQLN1 and UBQLN2, were down-regulated. These changes highlight how CVB3 disrupts antigen processing and presentation at multiple steps to evade detection by cytotoxic CD8^+^ T cells, echoing similar immune escape features previously observed in infected β cells (*9*).

Third, CVB3 disrupted type I interferon (IFN)–related pathways at multiple levels (Fig. 1G). Host sensors of viral RNA [protein kinase R (EIF2AK2), RNA helicase A (DHX9)] and self-DNA (DDX41) were down-regulated, indicating impaired recognition of viral replication intermediates and infection-induced cytosolic damage. TRIM56, a key type I IFN–inducing factor, was also reduced, suggesting that CVB3 suppresses IFN production pathways to limit host antiviral responses. Downstream, the IFN signaling axis was disrupted by the loss of signal transducer and activator of transcription 1 (STAT1), IFN-stimulated gene 15 (ISG15), and apoptosis-linked IFN-stimulated genes PDCD4 and FADD, further preventing immune clearance of infected cells. In addition, several translation regulatory factors (ELF1, EIF4G1/2/3, and YTHDF1/3) were down-regulated, consistent with a global reduction in host protein synthesis, which may, in turn, limit ISG expression. Suppression of multiple nucleoporins (RANBP2, NUP153, NUP188, NUP35, NUP62, and NUP214) was also evident, potentially disrupting the nuclear trafficking of transcription factors and further attenuating ISG expression. Collectively, these alterations suggest a coordinated viral strategy that disrupts each step of the IFN cascade: from nucleic acid sensing to IFN production and signaling, ultimately impairing ISG translation and facilitating CVB3 replication and spreading under minimal antiviral pressure.

Last, CVB3 infection remodeled autophagy pathways (Fig. 1H). Early autophagy initiators, including ATG13 and AMBRA1, were up-regulated, reflecting a host response to the increase of undegraded proteins and organelles caused by inhibited anterograde trafficking (Fig. 1F). This enhanced initiation may increase phagophore formation, providing CVB3 with scaffolds for assembling viral replication organelles (VROs). In parallel, the Golgi structural protein golgin A2 (GOLGA2) was down-regulated, a change known to drive Golgi disassembly and release of membrane fragments that are often used for VRO assembly (*21*–*23*). Despite enhanced autophagy initiation, CVB3 infection blocked downstream autophagic progression. Proteins involved in phagophore elongation (MAP1LC3B2 and GABARAPL2), cargo recognition [SQSTM1, CALCOCO2, galectin-8 (LGALS8), and BAG3], autophagosome maturation (CHMP4B and TSG101), vesicle transport (EXOC8, EXOC4, and VPS33A), and lysosomal fusion [VPS25, SNF8, HGS, syntaxin 17 (STX17)] were all down-regulated. This pattern suggests that, while autophagosome formation is triggered, autophagic flux may be arrested, preventing lysosomal degradation of viral components and restricting autophagy-dependent antigen presentation. This inhibition of the autophagy flux often drives cells to use alternative pathways to eliminate autophagosomes, notably by releasing their contents in the extracellular space (*24*–*26*). Consistent with this, surface cell exposure of the lysosomal marker lysosome-associated membrane protein 1 (LAMP1/CD107a) increased upon CVB3 infection, in CaCo2 enterocytes (Fig. 1I) and also in ECN90 β cells (Fig. 1J).

Collectively, proteome changes in CVB3-infected CaCo2 enterocytes reflect a viral strategy to promote immune silencing and efficient replication. Infected enterocytes adopt a nonproliferative yet polarized and functionally active, phenotype, concomitantly suppressing antigen processing and presentation, IFN signaling, and autophagy maturation. In addition, filopodia-mediated CVB transmission provides a supplementary mechanism to evade immune surveillance.

### CVB3 infection down-regulates HLA-I and up-regulates HLA-II antigen presentation in CaCo2 enterocytes

**Fig. 2.**
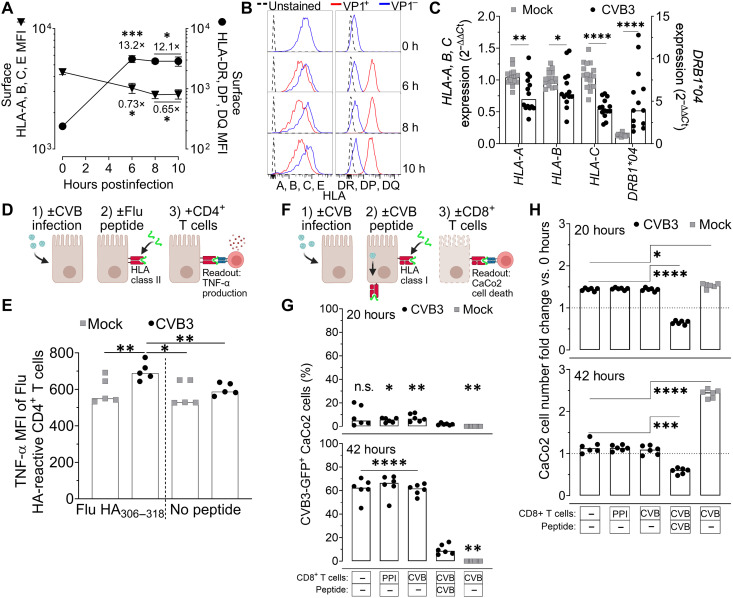
CVB3 down-regulates HLA-I and up-regulates HLA-II antigen presentation in CaCo2 enterocytes. (**A**) Flow cytometry MFI of surface HLA-I (left *y* axis) and HLA-II expression (right *y* axis) in CVB3-infected CaCo2 enterocytes (300 MOI; mean ± SD of three to four replicates, representative experiment performed in triplicate). *P ≤ 0.02 and ***P = 0.001 versus 0 hours postinfection by Student’s *t* test. (**B**) Representative histograms from the same experiments of surface HLA-I (left) and HLA-II (right) expression in infected (VP1^+^) versus noninfected (VP1^−^) CaCo2 enterocytes. (**C**) Median fold change of reverse transcription quantitative polymerase chain reaction (RT-qPCR) mRNA expression of *HLA-A*, *B*, *C*, and HLA-DRB1*04 in CVB3- versus mock-infected CaCo2 enterocytes (300 MOI, 6 hours; 12 replicates, representative experiment performed in triplicate). *P = 0.005, **P = 0.025, and ****P ≤ 0.0001 by Mann-Whitney *U* test. (**D**) Schematic of CD4^+^ T cell activation assays to assess HLA-DR*04:01 up-regulation. CVB3- or mock-infected CaCo2 enterocytes (10 MOI, 24 hours; median of five replicates, representative experiment performed in triplicate) were pulsed or not with influenza hemagglutinin (Flu HA)_306–318_ peptide and cocultured with a Flu HA–reactive CD4^+^ T cell clone. (**E**) CD4^+^ T cell activation measured by intracellular tumor necrosis factor–α (TNF-α) expression. *P = 0.048 and **P = 0.01 by Student’s *t* test. (**F**) Schematic of CD8^+^ T cell activation assays to assess HLA-A2 down-regulation. CVB3-GFP– or mock-infected CaCo2 enterocytes (10 MOI; median of six replicates, representative experiment performed in duplicate) were pulsed or not with CVB_1246–1254_ peptide and cocultured or not with irrelevant preproinsulin (PPI)_15–24_–reactive or cognate CVB-reactive primary CD8^+^ T cell receptor (TCR) transductants for live-cell imaging. (**G**) Percent CVB3-GFP^+^–infected CaCo2 enterocytes at 20 hours (top) and 42 hours (bottom). *P = 0.013, **P ≤ 0.004, and ****P ≤ 0.0001 by Student’s *t* test versus CVB T cells/CVB peptide condition. (**H**) Fold change at 20 hours (top) and 42 hours (bottom) versus 0 hours in the number of live (mKate2^+^) CaCo2 cells (transduced with the mKate2 nuclear marker). *P ≤ 0.014, ***P ≤ 0.0002, and ****P ≤ 0.0001 by Student’s *t* test, comparing the first three and the last two conditions.

Given the observed proteome downmodulation of antigen presentation and processing pathways in CVB3-infected CaCo2 enterocytes, we next assessed the modulation of surface HLA-I and HLA-II expression by flow cytometry. CVB3 infection induced a sustained dual effect (Fig. 2A): a reduction in HLA-I (∼1.5-fold decrease from 0 hpi) and an induction of HLA-II (∼13-fold increase). Stratifying CaCo2 enterocytes by infection status revealed that these changes were exclusively driven by the VP1^+^ subset, whereas VP1^−^ bystander cells remained unaffected (Fig. 2B). Similarly, significant down-regulation of *HLA-A*, *HLA-B*, and *HLA-C* and up-regulation of HLA-DRB1*04 transcripts were observed (Fig. 2C).

We next assessed whether the observed HLA-II up-regulation enhanced antigen presentation to CD4^+^ T cells. CaCo2 cells were either CVB3 or mock infected, pulsed or not with an influenza hemagglutinin (Flu HA)_306–318_ peptide, and cocultured with a DRB1*04:01-restricted, Flu HA_306–318_–reactive CD4^+^ T cell clone (Fig. 2D). CVB3-infected, peptide-pulsed CaCo2 cells elicited superior T cell activation than mock-infected controls, measured by intracellular tumor necrosis factor–α (TNF-α) staining (Fig. 2E), demonstrating the functional relevance of HLA-II up-regulation.

Similarly, we addressed the functional impact of HLA-I down-regulation by measuring CD8^+^ T cell cytotoxicity by live-cell imaging (Fig. 2F). CaCo2 cells transduced with the nuclear marker mKate2 were infected with CVB3-GFP (10 MOI) and pulsed or not with CVB3_1249–1257_ peptide (KLNSSVYSL, presented by CVB3-infected CaCo2 enterocytes; see Table 1) to enhance the endogenous viral peptide presentation. They were then cocultured with HLA-A2–restricted T cell receptor (TCR) transductants (*9*) recognizing either CVB3_1249–1257_ or an irrelevant [preproinsulin (PPI)_15–24_] peptide. GFP^+^ cells increased from 0 to 5% at 20 hpi (Fig. 2G, top), marking the onset of viral spreading, and reached 60% at 42 hpi (Fig. 2G, bottom). However, the presence of CVB3-reactive (or PPI-reactive) CD8^+^ T cells did not inhibit viral spreading, unless the peptide was exogenously added, confirming the efficient immune escape afforded by HLA-I down-regulation. Accordingly, upon CVB3 infection, CaCo2 cell numbers remained stable across conditions irrespective of T cell coculture in the absence of CVB3 peptide pulsing (Fig. 2H). At 42 hpi, CVB3-infected CaCo2 cell numbers decreased compared to the mock-infected condition, which underwent some proliferation. This is in line with the reduced expression of proliferation/regeneration-associated proteins upon CVB3 infection observed by proteomics (Fig. 1E).

**Table 1.** HLA-A*02:01 immunopeptidome eluted from the CaCo2 infection model. For each predicted binder, all overlapping length variants eluted are listed, with asterisks denoting noneluted length variants with better binding scores. Peptides displaying a predicted NetMHCpan 4.2 binding rank ≤ 4% (scores marked in bold) were selected for in vitro HLA-A*02:01 binding assays (using the 9- to 10-mer length variant with the best predicted binding; fig. S2) except for those that were previously eluted from CVB-infected β cells but did not validate for CD8^+^ T cell recognition (*9*) (second last column). Peptide PLLESQIAT (NetMHCpan rank: 8.49%) was included in binding assays as a negative control. HLA-A*02:01 binders were retained when scoring a ≥2.3 fold change versus dimethyl sulfoxide (DMSO) diluent in in vitro binding assays (scores marked in bold). Experimentally confirmed HLA-A*02:01 peptide binders were selected for T cell assays and are indicated by a “>” symbol in the first column. NA, not applicable.

CVB3 sequence	Start–end	Length	Number of hits	Predicted A*02:01 binding (NetMHCpan, % rank)	Experimental A*02:01 binding [fold change, half-life (min)]	Previous β cell elution, T cell validation	Viral protein
IMIKSLPALN	60–69	10	1/6	7.05	NA	Elution+, validation−	P1
> IMIKSLPAL*	60–68	9	NA	**0.11**	**4.0, 199.1**	No	P1
GLFGQNMQY	159–167	9	4/6	**2.21**	1.0, 28.2	Elution+, no validation	P1
> IVMPYTNSV	271–279	9	6/6	**0.04**	**3.7, 260.6**	**Elution+, validation+**	P1
AMATGKFLL	456–464	9	6/6	**0.36**	NA	Elution+, validation−	P1
MATGKFLL	457–464	8	5/6	18.23	NA		P1
AMLGTHVIWDV	480–490	11	2/6	1.65	NA	No	P1
> MLGTHVIWDV	481–490	10	3/6	**2.31**	**3.3, 144.7**	No	P1
LGTHVIWDV	482–490	9	2/6	11.74	NA	No	P1
GTHVIWDV	483–490	8	5/6	14.11	NA	No	P1
RIYFKPKHV	804–812	9	6/6	**0.67**	2.2, 98.0	Elution+, no validation	P2
GAFGQQSGAV	852–861	10	4/6	9.88	NA	No	P2
GQQSGAVYV*	855–863	9	NA	**0.83**	1.3, 147.7	No	P2
> GVKDYVEQL	1002–1010	9	1/6	**0.60**	**2.5, 100.8**	No	P2
PLLESQIAT	1154–1162	9	1/6	8.49	1.1, 182.6	No	P3
> KLNSSVYSL	1249–1257	9	2/6	**0.03**	**2.3, 194.1**	**Elution+, validation+**	P3
LEEKGILFTSPFVL	1306–1319	14	6/6	13.11	NA	No	P3
EEKGILFTSPFVL	1307–1319	13	4/6	17.87	NA	No	P3
ILFTSPFVL	1311–1319	9	5/6	**0.22**	NA	Elution+, validation−	P3
FTSPFVLA	1313–1320	8	1/6	10.62	NA	No	P3
SVGTTLEALFQ	1419–1429	11	1/6	54.16	NA	No	P3
SVGTTLEAL*	1419–1427	9	NA	**3.21**	1.0, 61.7	No	P3
TTLEALFQ	1422–1429	8	1/6	43.06	NA	No	P3
PRLKANFEEAIFSK	1771–1784	14	2/6	88.61	NA	No	P3
RLKANFEEA*	1772–1780	9	NA	**1.40**	1.5, 60.4	No	P3
YIGNVNTHVDE	1785–1795	11	1/6	30.70	NA	No	P3
> YIGNVNTHV*	1785–1793	9	NA	**0.19**	**3.2, 174.6**	No	P3
GLNLPMVTYV	1872–1881	10	1/6	**0.21**	1.5, 49.1	No	P3
GLNLPMVTY	1872–1880	9	5/6	6.63	NA	No	P3
> SLSPVWFA	1963–1970	8	3/6	**2.10**	**2.7, 93.1**	No	P3

Collectively, these results highlight a modest CVB3-mediated cytopathic effect but no CD8^+^ T cell–mediated cytotoxicity due to immune escape through down-regulation of HLA-I antigen presentation. In contrast, CD4^+^ T cell responses are amplified through HLA-II up-regulation.

### Both direct infection and viral antigen uptake lead to a limited presentation of HLA-bound CVB3 peptides focused on specific viral regions

**Fig. 3.**
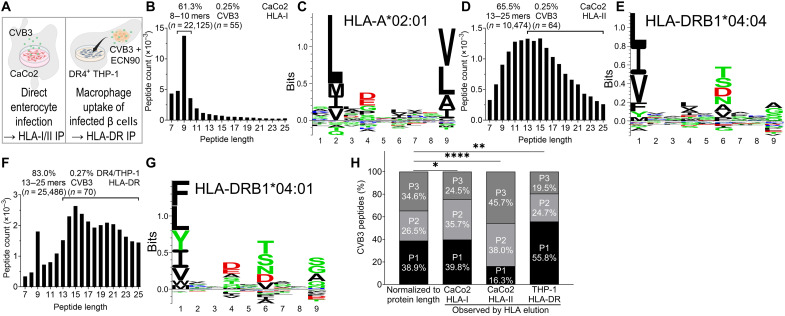
Both direct infection and viral antigen uptake lead to presentation of HLA-bound CVB3 peptides focused on specific viral regions. (**A**) Schematic of the in vitro infection used for immunopeptidomics studies. Left: Direct enterocyte infection (CaCo2 cells, CVB3: 300 MOI, 10 hours), followed by HLA-I immunoprecipitation (IP) [(B) and (C)] and HLA-II IP [(D) and (E)]. Right: Macrophage uptake of infected β cells (DR4/THP-1 macrophages cocultured with apoptotic CVB3-infected ECN90 β cells: 10 MOI, 72 hours), followed by HLA-DR IP [(F) and (G)]. (**B**) Length distribution of HLA-I–bound peptides eluted from CVB3-infected CaCo2 enterocytes (six biological replicates, 227 to 307 × 10^6^ cells per each). Bars represent cumulative counts of unique peptides from all replicates. Number and percentage of expected 8- to 10-mer peptides and of CVB3-derived peptides within this length range are indicated. (**C**) Predominant binding motif of eluted unique peptides visualized by MHCMotifDecon, consistent with an HLA-A*02:01–binding motif. The *x* axis indicates the residue position within the 9-mer core sequence. Each amino acid is represented by its single-letter code, with its size proportional to its frequency at the indicated position. (**D** and **E**) Length distribution of HLA-II–bound peptides (D) and predominant HLA-DRB1*04:04–like binding motif of eluted unique peptides (E) from CVB3-infected CaCo2 enterocytes. Replicates and data representation are the same as in (B) and (C). (**F** and **G**) Length distribution of HLA-DR–bound peptides (F) and predominant HLA-DRB1*04:01–like binding motif of eluted unique peptides (G) from DR4/THP-1 macrophages exposed to CVB3-infected ECN90 β cells (four biological replicates, 14 to 15 × 10^6^ cells per each). Data representation is the same as in (B) and (C). (**H**) Percent distribution across the structural P1 and nonstructural P2 and P3 viral proteins of HLA-eluted unique CVB3 peptides (infected CaCo2 cells, second and third bar; DR4/THP-1, fourth bar) versus those expected based on the amino acid length of each protein (first bar). *P = 0.048, **P = 0.004, and ****P < 0.0001 by χ^2^ test.

Given the modulation of HLA-I and HLA-II pathways in CVB3-infected CaCo2 enterocytes, we next aimed to define the HLA-displayed viral peptides using high-resolution mass spectrometry. To this end, we used two complementary infection models (Fig. 3A). First, CVB3-infected CaCo2 enterocytes to model the primary antigen presentation at the gut entry site, and second, CVB3-infected ECN90 β cells, which, upon apoptosis, were allowed to be taken up by DRB1*04:01-transduced THP-1 (DR4/THP-1) monocytic cell line differentiated into macrophages, to model the secondary antigen presentation at the pancreas target site.

In the primary antigen presentation model of CVB3-infected CaCo2 enterocytes, the HLA-I dataset obtained by immunoprecipitation (IP) with the pan–HLA-I Ab W6/32 yielded the expected 8- to 10-mer peptide length distribution, with a dominant peak at nine amino acids (Fig. 3B). Of the 22,125 unique 8- to 10-mer sequences eluted, 55 (0.25%) were CVB3 derived. The main peptidome motif was consistent with the canonical HLA-A2 hydrophobic anchor residues at position 2 (P2) [leucine (L), methionine (M), isoleucine (I), and valine (V)] and at the C-terminal P9 [V, L, alanine (A), and I] (Fig. 3C). Focusing on HLA-A2–restricted peptides, most (^7^/_13_; 54%) eluted CVB3 peptides predicted to bind HLA-A2 (Table 1) and experimentally tested in vitro were confirmed as binders (fig. S2). Among those, five unknown HLA-A2–restricted peptides not previously detected in CVB-infected β cell immunopeptidomes (*9*) were identified and retained for further CD8^+^ T cell studies: CVB3_60–68_, CVB3_481–490_, CVB3_1002–1010_, CVB3_1785–1793_, and CVB3_1963–1970_ (sequence alignment with other CVB serotypes presented in table S1). Several HLA-A2 ligands previously eluted from β cells (*9*) were also identified in CaCo2 enterocytes, including two previously validated for CD8^+^ T cell recognition: CVB3_271–279_ and CVB3_1249–1257_ (previously annotated as CVB1_1246–1254_; 100% identity across serotypes).

The HLA-II dataset obtained from CVB3-infected CaCo2 enterocytes by IP with the pan–HLA-II Ab IVA12 displayed the expected length distribution skewed toward peptides ≥13 amino acids long (Fig. 3D) (*27*); 64 (0.25%) of the 10,474 unique 13- to 25-mer sequences eluted were CVB3 derived. The main peptidome motif was consistent with an HLA-DRB1*04:04 restriction: P1 harboring hydrophobic (L, I, V, and M) or aromatic residue [phenylalanine (F) and tyrosine (Y)]; polar [threonine (T), serine (S), aspartic acid (D), and asparagine (N)] or small residue (A and V) at P6; and heterogeneity at P9 (Fig. 3E).

Similarly, the secondary DR4/THP-1 macrophage antigen presentation model yielded an immunopeptidome (using the anti–HLA-DR Ab L243) enriched in long (≥13 amino acids) sequences (Fig. 3F). Of the 25,486 unique 13- to 25-mer sequences eluted, 70 (0.27%) were CVB3 derived, with an overall predominant DRB1*04:01 motif (Fig. 3G): similar to DRB1*04:04 at P1 and P6 and with hydrophobic or small [S, glycine (G), and A] or polar residue (Q and N) at P9. This high motif similarity between DRB1*04:04 and DRB1*04:01 is consistent with the reported peptide binding promiscuity of these two allotypes (*28*). We therefore also considered predicted DRB1*04:04 binders for downstream HLA-DRB1*04:01 prediction and experimental binding analyses. Most (^5^/_6_; 83%) eluted CVB3 peptides predicted to bind HLA-DRB1*04:01 (Table 2) were experimentally confirmed in vitro using T cell competition binding assays (fig. S3): CVB3_29–44_, CVB3_256–270_, CVB3_889–902_, CVB3_1431–1445_, and CVB3_1620–1634_.

**Table 2.** HLA-DRB1*04:01 immunopeptidome eluted from the CaCo2 and DR4/THP-1 infection models. For each predicted binder, all overlapping length variants eluted are listed, with asterisks denoting noneluted length variants with better binding scores. Peptides displaying a predicted NetMHCIIpan binding rank ≤ 4% (scores marked in bold; predicted DRB1*04:04 binding in parenthesis for peptides eluted from CaCo2 enterocytes) were selected for in vitro HLA-DRB1*04:01 binding assays (using the 14- to 16-mer length variant with the best predicted binding). Peptides VKDYVEQLGNAFGSG and IATIEQSAPSQSDQE (NetMHCIIpan rank: 7.49 and 6.07%, respectively) were included in binding assays as negative controls. HLA-DRB1*04:01 binders were retained when scoring a median inhibitory concentration (IC_50_) < 400 μM versus DMSO diluent in in vitro binding assays (scores marked in bold). Experimentally confirmed HLA-DRB1*04:01 peptide binders were selected for T cell assays and are indicated by a > symbol in the first column.

CVB3 sequence	Start–end	Length	CaCo2 or DR4/THP-1 hits	Number of hits	Predicted DRB1*04:01 binding (NetMHCIIpan, % rank)	Experimental DRB1*04:01 binding (IC_50_, μM)	Viral protein
HYTNINYYKDAASNSA	26–41	16	DR4/THP-1	^1^/_4_	0.98		P1
HYTNINYYKDAASNS	26–40	15	DR4/THP-1	^1^/_4_	2.90		P1
YTNINYYKDAASNSA	27–41	15	DR4/THP-1	^4^/_4_	0.65		P1
YTNINYYKDAASNS	27–40	14	DR4/THP-1	^4^/_4_	2.47		P1
TNINYYKDAASNSANRQD	28–45	18	DR4/THP-1	^1^/_4_	0.15		P1
TNINYYKDAASNSANR	28–43	16	DR4/THP-1	^2^/_4_	0.08		P1
NINYYKDAASNSANRQD	29–45	17	DR4/THP-1	^4^/_4_	0.10		P1
> NINYYKDAASNSANRQ	29–44	16	DR4/THP-1	^4^/_4_	**0.06**	**222.6**	P1
NINYYKDAASNSANR	29–43	15	DR4/THP-1	^4^/_4_	0.06		P1
NINYYKDAASNSAN	29–42	14	DR4/THP-1	^1^/_4_	0.14		P1
INYYKDAASNSANRQD	30–45	16	DR4/THP-1	^4^/_4_	0.12		P1
INYYKDAASNSANRQ	30–44	15	DR4/THP-1	^4^/_4_	0.07		P1
INYYKDAASNSANR	30–43	14	DR4/THP-1	^4^/_4_	0.09		P1
INYYKDAASNSAN	30–42	13	DR4/THP-1	^3^/_4_	0.27		P1
NYYKDAASNSANRQD	31–45	15	DR4/THP-1	^2^/_4_	0.33		P1
NYYKDAASNSANRQ	31–44	14	DR4/THP-1	^4^/_4_	0.21		P1
NYYKDAASNSANR	31–43	13	DR4/THP-1	^4^/_4_	0.36		P1
YYKDAASNSANRQ	32–44	13	DR4/THP-1	^3^/_4_	2.04		P1
AGMGVGVGNLTIFPHQWINLR	244–264	21	DR4/THP-1	^1^/_4_	88.46		P1
> FPHQWINLRTNNSAT	256–270	15	DR4/THP-1	^2^/_4_	**0.27**	**109.8**	P1
> RDLLVSTTTAHGCD	889–902	14	DR4/THP-1	^4^/_4_	**0.89**	**306.1**	P2
GVKDYVEQLGNAFGSG	1002–1017	16	CaCo2	^6^/_6_	9.18 (0.80)		P2
GVKDYVEQLGNAF	1002–1014	13	CaCo2	^1^/_6_	38.52 (62.67)		P2
VKDYVEQLGNAFGSG*	1003–1017	15	CaCo2	NA	7.49 (0.50)	1109.0	**P2**
DYVEQLGNAFGSGFTNQ	1005–1021	17	CaCo2	^1^/_6_	39.65 (9.17)		P2
LKQLPLLESQIATIEQSAP	1150–1168	19	CaCo2	^1^/_6_	62.20 (45.14)		P3
LLESQIATIEQSAP	1155–1168	14	CaCo2	^2^/_6_	48.32 (43.88)		P3
QIATIEQSAPSQSDQEQ	1159–1175	17	CaCo2	^1^/_6_	8.05 (4.95)		P3
IATIEQSAPSQSDQE*	1160–1174	15	CaCo2	NA	6.07 (3.85)	598.7	**P3**
SQSDQEQLFSNVQYFAHY	1169–1186	18	CaCo2	^2^/_6_	68.53 (47.73)		P3
SQSDQEQLFSNVQYFAH	1169–1185	17	CaCo2	^4^/_6_	66.32 (40.92)		P3
SQSDQEQLFSNVQY	1169–1182	14	CaCo2	^4^/_6_	67.22 (39.47)		P3
QQAVVIMDDLCQNPDG	1269–1284	16	DR4/THP-1	^1^/_4_	4.10		P3
AVVIMDDLCQNPDG	1271–1284	14	DR4/THP-1	^1^/_4_	20.58		P3
DLCQNPDGKDVSLFCQMVSSVDF	1277–1299	23	DR4/THP-1	^1^/_4_	93.80		P3
GPPVYREIKISVAPETPPPPAIADL	1430–1454	25	CaCo2	^6^/_6_	7.08 (8.26)		P3
GPPVYREIKISVAPETPPPPAIAD	1430–1453	24	CaCo2	^2^/_6_	7.26 (8.50)		P3
>––PPVYREIKISVAPET*	1431–1445	15	CaCo2	NA	**0.74 (5.30)**	**192.2**	P3
YREIKISVAPETPPP*	1434–1448	15	CaCo2	NA	4.06 (2.70)		P3
IKISVAPETPPPPAIADL	1437–1454	18	CaCo2	^6^/_6_	37.23 (29.88)		P3
IKISVAPETPPPPAIAD	1437–1453	17	CaCo2	^1^/_6_	29.38 (23.18)		P3
KISVAPETPPPPAIADL	1438–1454	17	CaCo2	^3^/_6_	34.71 (30.69)		P3
ISVAPETPPPPAIADLLK	1439–1456	18	CaCo2	^5^/_6_	76.65 (73.86)		P3
ISVAPETPPPPAIADL	1439–1454	16	CaCo2	^6^/_6_	55.29 (54.87)		P3
VAPETPPPPAIADLLK	1441–1456	16	CaCo2	^1^/_6_	94.90 (97.65)		P3
VAPETPPPPAIADL	1441–1454	14	CaCo2	^5^/_6_	93.39 (97.38)		P3
AIADLLKSVDSEAVREY	1450–1466	17	CaCo2	^1^/_6_	3.80 (2.18)		P3
IADLLKSVDSEAVRE*	1451–1465	15	CaCo2	NA	**2.49 (1.39)**	737.1	P3
RNEKFRDIRGFLAKEE	1619–1634	16	DR4/THP-1	^4^/_4_	2.04		P3
> NEKFRDIRGFLAKEE*	1620–1634	15	DR4/THP-1	NA	**1.62**	**65.4**	P3
FRDIRGFLAKEEVEV	1623–1637	15	DR4/THP-1	^1^/_4_	43.95		P3
RGFLAKEEVEVNE	1627–1639	13	DR4/THP-1	^1^/_4_	17.62		P3

Mapping of the CVB3-derived peptides identified to structural (P1) and nonstructural (P2 and P3) regions of the viral polyprotein (Fig. 3H) revealed a skewed distribution specific to each in vitro model. In the primary antigen presentation model of infected CaCo2 enterocytes, the percentage of HLA-eluted peptides mapped significantly more than expected to nonstructural regions (based on their relative aa length): P2 for HLA-A2, as we previously observed in ECN90 β cells (*9*); both P2 and P3 for HLA-DRB1*04:04. In contrast, the secondary antigen presentation model of DR4/THP-1 macrophages yielded an enrichment for peptides derived from the structural region (P1) that builds up mature virions, consistent with a dominant mechanism of exogenous uptake of viral material rather than endogenous viral replication. No open reading frame (ORF)–translated peptides were detected.

Collectively, both CVB3-infected CaCo2 enterocytes and DR4/THP-1 macrophages exposed to infected β cells display a limited repertoire of CVB3-derived peptides via both HLA-I and HLA-II. These peptides are predominantly derived from nonstructural viral proteins in infected cells and from structural proteins upon endocytosis of viral material. This immunopeptidome expands the HLA-A2–restricted one previously identified in CVB-infected β cells (*9*) and provides a previously unknown HLA-DRB1*04:01–restricted immunopeptidome for CD4^+^ T cell studies.

### The immunopeptidome of CVB3-infected human duodenal organoids recapitulates findings in CaCo2 enterocytes

**Fig. 4.**
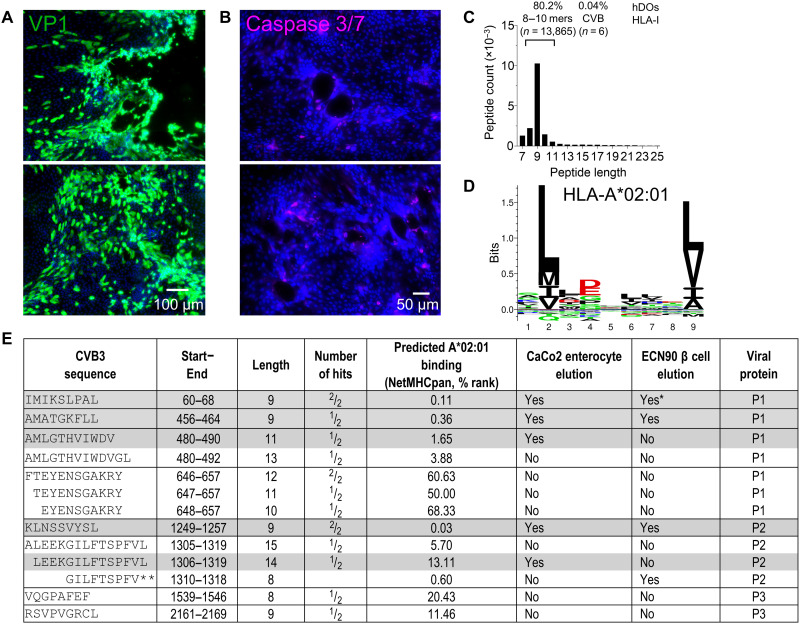
HLA-A2–restricted peptides eluted from CVB3-infected hDOs. HLA-A2^+^ hDOs were infected with CVB3 (100 MOI; 15 hours), followed by HLA-I IP. (**A**) Immunofluorescence staining for VP1 (green), with nuclei counterstained in blue. Images are adjacent vision fields from the same hDO specimen. (**B**) Immunofluorescence staining for activated caspase-3/7 (purple). (**C**) Length distribution of HLA-I–bound peptides eluted from CVB3-infected hDOs. One biological replicate (∼10^7^ cells) with two technical replicates was acquired, and unique peptides across replicates were counted. Number and percentage of 8- to 10-mer peptides and of CVB3-derived peptides within this length range are indicated. (**D**) Predominant binding motif of eluted unique peptides visualized by MHCMotifDecon, consistent with an HLA-A*02:01–binding motif. The *x* axis indicates the residue position within the 9-mer core sequence. Each amino acid is represented by its single-letter code, with its size proportional to its frequency at the indicated position. (**E**) Eluted HLA-A*02:01 immunopeptidome. For each predicted binder, all overlapping length variants eluted are listed. Peptides eluted from CVB3-infected CaCo2 enterocytes (third last column) are highlighted in gray. Peptides previously eluted from CVB-infected ECN90 β cells are indicated in the second last column. *Peptide eluted as a longer CVB3_60–69_ length variant. **Noneluted length variant with better binding score.

To confirm the immunopeptidomics results obtained in the primary antigen presentation model of CVB3-infected CaCo2 enterocytes in a more physiological setting, we infected HLA-A2^+^ human duodenal organoids (hDOs) with CVB3 at a lower MOI of 100. As in the CaCo2 model, despite widespread infection (Fig. 4A; mean ± SD: 44.6 ± 16.8% VP1^+^ cells), hDOs remained largely viable (Fig. 4B; 4.6 ± 3.3% activated caspase-3/7^+^ cells). We identified 17,286 unique HLA-I ligands, with 8 to 10 mers accounting for 80.2% of these peptides, of which 0.04% were CVB3 derived (Fig. 4C). HLA-A*02:01 was the most represented binding motif (Fig. 4D). Among these HLA-A*02:01–restricted CVB3 peptides (Fig. 4E), three were shared with those eluted from CaCo2 enterocytes and ECN90 β cells: two from the structural P1 region (CVB3_60–68_ and CVB3_456–464_) and one from the nonstructural P2 region (CVB3_1249–1257_). Other two P2 peptides (CVB3_480–490_ and CVB3_1306–1319_) appeared exclusively in enterocytes, both CaCo2 and hDOs, confirming cell-type–biased presentation. When grouping into nested peptide sets (*n* = 8), ^5^/_8_ (62.5%) CVB3 peptides eluted from hDOs were also identified in CaCo2 enterocytes despite the more limited peptide diversity captured by the lower cell input available for hDOs. Collectively, these findings validate the in vitro CaCo2 infection model used in previous experiments.

### CVB3-reactive CD8^+^ T cells display naïve- and exhausted effector/memory-like phenotypes

The panel of HLA-A2–restricted CVB3 candidate epitopes obtained by immunopeptidomics provided a toolkit to track their cognate CD8^+^ T cells. To this end, circulating CD8^+^ T cells from six CVB-seropositive HLA-A2^+^ (A*02:01^+^) healthy donors (table S2) were analyzed using a combinatorial HLA-A2 multimer (MMr) staining strategy (fig. S4, A and B) (*9*, *29*–*32*). The peptide panel included both previously unknown candidates and two epitopes (CVB3_271–279_ and CVB3_1249–1257_) identified both herein and previously from CVB-infected β cells and previously validated for CD8^+^ T cell recognition (*9*). Control Flu matrix protein (MP)_58–66_ and MelanA_26–35_ epitopes were included to provide a benchmark for effector/memory and naïve phenotypes, respectively.

**Fig. 5.**
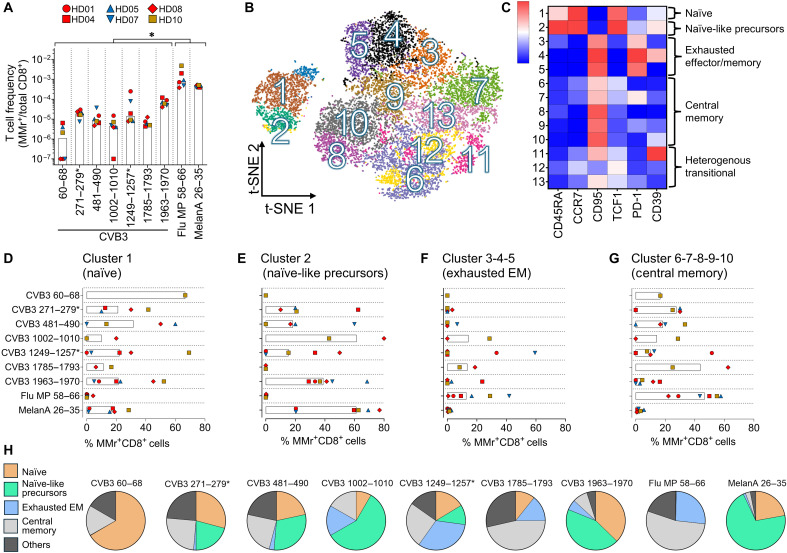
Naïve- and exhausted effector/memory-like phenotypes in CVB3-reactive CD8^+^ T cells. (**A**) Blood frequency of MMr^+^CD8^+^ T cells recognizing the indicated CVB3 peptides in CVB-seropositive HLA-A*02:01^+^ healthy donors (*n* = 6). The frequency of Flu MP_58–66_ and MelanA_26–35_ peptide-reactive CD8^+^ T cells included as controls for effector/memory and naïve phenotypes, respectively, is shown for comparison. Data points with <5 MMr^+^ cells counted were excluded. The CVB3_271–279_ and CVB3_1249–1257_ epitopes previously identified in β cell immunopeptidomes and validated for T cell recognition are indicated by asterisks. Each symbol represents a donor, and bars indicate median values. *P = 0.031 for the individual comparison of each CVB3 MMr^+^ frequency with Flu or MelanA MMr^+^ frequency by Wilcoxon signed-rank test. (**B**) t-SNE projection of MMr^+^CD8^+^ T cells, clustered on the basis of the expression of the indicated phenotypic markers of T cell differentiation (CD45RA, CCR7, CD95, and TCF1) and activation/exhaustion (PD-1 and CD39). Each dot represents a single cell. (**C**) Heatmap of normalized expression intensity of each phenotypic marker across the 13 clusters. (**D** to **G**) Percent MMr^+^CD8^+^ T cells across clusters. Each symbol represents a donor, and bars indicate median values. (**H**) Cumulative relative cluster distribution in MMr^+^CD8^+^ T cells reactive to the indicated peptides.

Barring CVB3_60–68_, cognate MMr^+^CD8^+^ T cells were detectable in all donors for the entire set of CVB3 peptides (Fig. 5A; except CVB3_1002–1010_ for donor HD04), thus validating them as target epitopes. However, their frequencies were overall low, with median values ranging from 5 to 70/10^6^ CD8^+^ T cells, i.e., ∼10- to 100-fold lower than what observed for Flu and MelanA epitope-reactive CD8^+^ T cells (median frequency of 815 and 445/10^6^ CD8^+^ T cells, respectively). More robust yet overall weak responses were detected for the newly identified epitope CVB3_1963–1970_ (median frequency of 59/10^6^ CD8^+^ T cells).

We next examined the phenotypic composition of these epitope-reactive populations by projecting all MMr^+^CD8^+^ T cells into a t-distributed stochastic neighbor embedding (t-SNE) map generated from multiparametric flow cytometry data (Fig. 5B and representative staining in fig. S4C). This allowed us to visualize their distribution across 13 clusters defined by differentiation [CD45RA, CCR7, CD95, and T cell factor 1 (TCF1)] and activation/exhaustion markers [programmed cell death protein 1 (PD-1) and CD39] (Fig. 5C). Cluster 1 displayed a naïve phenotype (CD45RA^+^CCR7^+^CD95^−^TCF1^+^). Cluster 2 displayed the same naïve-like homing phenotype yet coexpressed CD39 and, to a lesser extent, PD-1, indicating precursors with features of chronic activation. Clusters 3 to 5 corresponded to effector/memory T cells (CD45RA^−^CCR7^−^CD95^hi^TCF1^−^) with exhaustion features (PD-1^hi^ with variable CD39 expression). In comparison, clusters 6 to 10 reexpressed some CCR7 and were CD95^int^TCF1^lo^, corresponding to central memory CD8^+^ T cells. Clusters 11 to 13 had more heterogenous, transitional phenotypes.

The distribution of epitope reactivities within these clusters revealed distinct patterns (Fig. 5, D to H). Barring CVB3_60–68_ due to its low cognate T cell frequencies, most epitope reactivities (CVB3_271–279_, CVB3_481–490_, CVB3_1002–1010_, and CVB3_1963–1970_) were dominated by naïve and naïve-like precursors, while CVB3_1249–1257_ featured higher proportions of exhausted effector/memory CD8^+^ T cells. The representation of central memory clusters was overall low across all CVB3 reactivities, barring their unique dominance for the newly identified CVB3_1785–1793_ epitope yet also recognized at low frequencies (Fig. 5A). The control Flu MP_58–66_- and MelanA_26–35_–reactive CD8^+^ T cells displayed the expected predominant central memory and naïve-like phenotypes, respectively. Collectively, while some CVB3-reactive CD8^+^ T cells progress from a naïve to an activated naïve-like phenotype, this progression is limited by the development of exhaustion features, preventing their differentiation into central memory subsets.

### CVB3-reactive CD4^+^ T cells exhibit robust memory and T follicular helper phenotypes

The limited CVB3 antiviral responses measured in the CD8^+^ T cell compartment beg the question of whether these responses may rely predominantly on CD4^+^ T cells. To address this possibility, we evaluated CD4^+^ T cell responses in CVB-seropositive HLA-DRB1*04:01^+^ healthy donors (table S2), using the HLA-DRB1*04:01–restricted peptides identified by immunopeptidomics and the Flu HA_306–318_ epitope as a benchmark for effector/memory responses. Following a 24-hour stimulation of peripheral blood mononuclear cells (PBMCs) with each individual peptide, CD4^+^ T cells were analyzed by flow cytometry for the expression of activation-induced marker (AIM) signatures (*33*), comprising CD40L, 4-1BB, CD25, CD69, HLA-DR, OX40, and PD-L1. For each donor/peptide combination, after gating on activated CD40L^+^CD4^+^ T cells (fig. S5A), all 64 possible AIM combinations were assessed (i.e., CD40L alone or in combination with 1, 2, 3, 4, 5, or 6 other AIMs; fig. S5B). To achieve maximal specificity and sensitivity, the combinations retained for each donor were those that (i) did not yield any AIM^+^CD4^+^ T cell following the dimethyl sulfoxide (DMSO) negative control stimulation and (ii) did yield AIM^+^CD4^+^ T cells following each peptide stimulation analyzed individually (fig. S5, C and D).

**Fig. 6.**
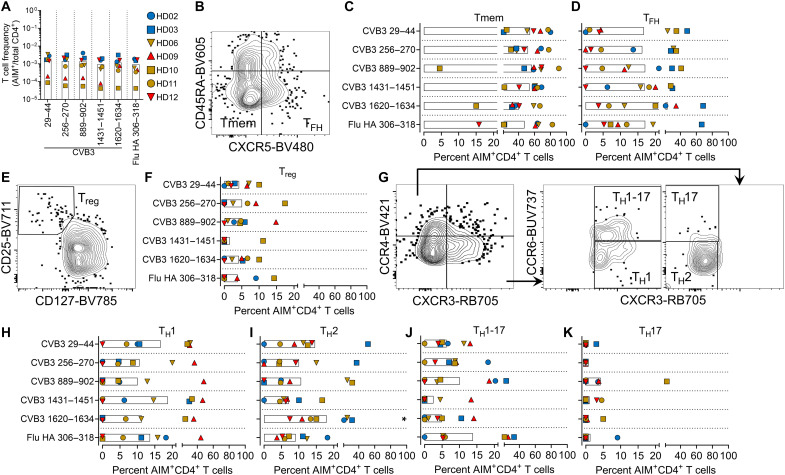
CVB3-reactive CD4^+^ T cells exhibit robust memory and T_FH_ phenotypes. (**A**) Blood frequency of AIM^+^CD4^+^ T cells recognizing the indicated CVB3 peptides after a 24-hour peptide stimulation in CVB-seropositive HLA-DRB1*04:01^+^ healthy donors (*n* = 7). The frequency of Flu HA_306–318_–reactive CD4^+^ T cells included as effector/memory control is shown for comparison. (**B**) Gating strategy to identify effector/memory (Tmem; CD45RA^−^CXCR5^−^) and T_FH_ (CD45RA^−^CXCR5^+^) subsets within AIM^+^CD4^+^ T cells. (**C** and **D**) Percentages of Tmem (C) and T_FH_ (D) subsets within AIM^+^CD4^+^ T cells reactive to each peptide. (**E**) Gating strategy to identify T_reg_ cells (CD25^high^CD127^low^) within AIM^+^CD4^+^ T cells. (**F**) T_reg_ percentages within AIM^+^CD4^+^ T cells. (**G**) Gating strategy to identify T helper (T_H_) polarization based on CCR4, CXCR3, and CCR6 expression within AIM^+^CD4^+^ T cells: T_H_1 (CXCR3^+^CCR4^−^CCR6^−^), T_H_2 (CXCR3^−^CCR4^+^CCR6^−^), T_H_17 (CXCR3^−^CCR4^+^CCR6^+^) and T_H_1/T_H_17 (CXCR3^+^CCR4^−^CCR6^+^). (**H** to **K**) Percentages of T_H_1 (H), T_H_2 (I), T_H_1/T_H_17 (J), and T_H_17 (K) subsets within AIM^+^CD4^+^ T cells. Each symbol represents a donor, and bars indicate mean values. *P = 0.016 for pairwise comparison with Flu HA–reactive AIM^+^CD4^+^ T cells by Wilcoxon signed-rank test.

In all donors, CVB3-reactive AIM^+^CD4^+^ T cells were readily detectable for all peptides tested, generally clustering together within a 10^−3^ frequency range (i.e., ∼1000 peptide-reactive T cells per million CD4^+^ T cells), comparable to the frequency measured for the recall control Flu HA_306–318_ epitope (Fig. 6A). While donors HD09 and HD10 displayed lower frequencies, the magnitude of CVB3-reactive responses also paralleled that for Flu HA_306–318_ in donor HD10. Overall, this validates the CVB3 peptides selected by immunopeptidomics as epitopes robustly targeted by CD4^+^ T cells.

To define functional phenotypes, we first analyzed AIM^+^CD4^+^ T cells for their CD45RA and CXCR5 expression to define conventional memory T cells (Tmem cells; CD45RA^−^CXCR5^−^) and T follicular helper cells (T_FH_ cells; CD45RA^−^CXCR5^+^) (Fig. 6B). Overall, more than 40% of CVB3-reactive CD4^+^ T cells displayed a Tmem phenotype, again comparable to that of control Flu HA–reactive CD4^+^ T cells (Fig. 6C). T_FH_ cells accounted for an average of more than 16% of epitope-reactive CD4^+^ T cells for both CVB3 and Flu HA (Fig. 6D). In contrast, regulatory T cells (T_reg_ cells; CD25^hi^CD127^lo/neg^; Fig. 6E) were less frequent (<5%) in most instances (Fig. 6F), and their fractions were inversely correlated with T cell frequencies [Spearman correlation coefficient (*r*) = −0.412, *P* = 0.007; fig. S5E]. These findings suggest that CVB3-reactive CD4^+^ T cells mount a robust memory and provide effective T_FH_ support, enabling the generation and maintenance of antiviral Ab responses.

We expanded our analysis to determine the T helper (T_H_) polarization of AIM^+^CD4^+^ T cells. T_H_ subsets were defined using CCR4, CXCR3, and CCR6 expression (Fig. 6G): T_H_1 (CXCR3^+^CCR4^−^CCR6^−^), T_H_2 (CXCR3^−^CCR4^+^CCR6^−^), T_H_17 (CXCR3^−^CCR4^+^CCR6^+^), and T_H_1/T_H_17 (CXCR3^+^CCR4^−^CCR6^+^). All CVB3 peptides elicited detectable T_H_1, T_H_2, and T_H_1/T_H_17 responses in most but not all donors (Fig. 6, H to J), with no statistically significant donor-paired differences compared to Flu HA. The only exception was CVB_1620–1634_, which showed a higher T_H_2 polarization compared to Flu HA. T_H_17 frequencies were low or undetectable across all epitopes (Fig. 6K).

Collectively, these results show that CVB3-reactive CD4^+^ T cells mount robust, polyfunctional memory responses, comparable to those elicited by Flu HA. This capacity contrasts with the limited effector/memory differentiation of CVB3-reactive CD8^+^ T cells.

## DISCUSSION

Understanding how immunity against CVB3 is shaped at the intestinal entry site could provide crucial insights into the mechanisms driving the onset of islet autoimmunity in T1D. This standing question has direct clinical relevance, as multiple studies have detected enteroviral persistence in the gut of T1D patients, implicating early mucosal immune responses as potential triggers of downstream β cell damage (*4*, *8*, *15*). We addressed this question by focusing on antigen presentation, the main gatekeeper of T cell–mediated immunity. While our previous work (*9*) focused on the dynamics of T cell–mediated cytotoxicity following viral antigen presentation by infected β cells, in the present work, we considered both the earliest intestinal location of viral entry and the later events following death of virus-infected cells, including β cells, and uptake by macrophages. To this end, we used two complementary infection models that recapitulate these routes of antigen presentation: an enterocyte model of direct infection and a macrophage model of indirect antigen uptake. These models combined the analysis of the host proteome, HLA-bound viral peptidome and the ensuing T cell responses to provide a comprehensive view of antiviral immune recognition.

At the proteomic level, we observed remodeling of multiple pathways that collectively impair antigen processing and presentation, alongside broad suppression of innate immune sensing. These changes are in agreement with the known activity of viral proteases (*34*), which cleave key adaptor proteins in the RIG-I–like and Toll-like receptor signaling pathways (*35*), disrupt ER-to-Golgi trafficking, and hinder HLA-I peptide loading (*36*–*38*). CVB3 also halted cell proliferation, which may redirect host biosynthetic resources toward viral replication (*39*), lowering protein turnover and, in turn, immune recognition (*40*, *41*). Alterations in autophagy-related proteins suggested increased early autophagic events but inhibition of late maturation and lysosomal fusion steps, a pattern reported for other Enteroviruses that exploit autophagy to generate replication membranes while restricting the degradative flux (*42*). As in β cells (*9*), live-cell imaging further revealed filopodia-mediated viral transmission from infected enterocytes. Nonlytic CVB shedding has been previously reported, either via membrane protrusions or autophagosome-derived vesicles, both of which can shield virions from immune recognition and Ab-mediated neutralization (*43*, *44*). These findings point to multiple, converging CVB3 immune escape strategies.

Guided by these proteomic signatures, surface protein expression and functional assays confirmed a reduction of surface HLA-I expression. This resulted into downstream inhibition of antiviral CD8^+^ T cell recognition and impaired killing of infected CaCo2 enterocytes, which were instead killed when the cognate CVB3 peptide was exogenously added. This suggests that CVB3 specifically limits exposure of its own peptides on top of HLA-I down-regulation. Limited T cell cytotoxicity was also previously observed in β cells, which were rather killed by the viral cytopathic effects (*9*), suggesting a conserved immune evasion mechanism across different target tissues. Unexpectedly, infected enterocytes further revealed an up-regulation of HLA-II, which resulted in an opposite outcome of enhancing CD4^+^ T cell recognition. Thus, the net effect of direct antigen presentation in infected enterocytes is a viral escape from CD8^+^ T cell cytotoxic surveillance and preservation of CD4^+^ T cell responses.

Looking at the CVB3 antigen presentation landscape, the HLA-A2– and HLA-DRB1*04:01–restricted immunopeptidome displayed by enterocytes was narrow and predominantly composed of nonstructural proteins, as reported for the HLA-I immunopeptidome of infected β cells (*9*). This may be expected in the context of productive infection and active intracellular viral replication because nonstructural proteins do not leave the cell with the release of mature virions but are eventually catabolized intracellularly, thus providing more accessible substrates for antigen presentation. In contrast, the HLA-DRB1*04:01–restricted immunopeptidome eluted from the nonreplicative macrophage model was focused on structural proteins, consistent with the uptake and processing of intact virions and viral debris. From these datasets, we validated previously unknown HLA-I–eluted (*n* = 4) and HLA-II–eluted (*n* = 5) CVB3 peptides as HLA-A2– and HLA-DRB1*04:01–binding epitopes targeted by CD8^+^ and CD4^+^ T cells, respectively. This panel further included two HLA-A2–restricted epitopes (CVB3_271–279_ and CVB3_1249–1257_) eluted from both infected enterocytes and β cells and previously validated as the immunodominant targets of CD8^+^ T cells (*9*). These reference epitopes were recognized by CD8^+^ T cells at low frequencies, similar to those observed for the novel ones identified.

This more comprehensive epitope panel afforded greater granularity to analyze anti-CVB3 cellular immunity than previously obtained from the β cell peptidome, notably by tracking both CD8^+^ and CD4^+^ T cells. Expanding our previous observation (*9*) of high circulating naïve CD8^+^ T cell fractions, we here confirm that CVB3-reactive CD8^+^ T cell responses display low frequencies, reflecting early differentiation states comprising both naïve- and effector/memory-like phenotypes with exhaustion features. These phenotypes included a pool of naïve-like precursors that retain lymphoid homing markers but have acquired chronic activation/exhaustion markers (PD-1, and CD39), likely as a result of repeated or persistent exposure to CVB antigens. On one hand, this predominance of circulating early-differentiated intermediates may reflect the preferential retention of effector/memory fractions within lymphoid organs (*9*). On the other, suboptimal CD8^+^ T cell priming and early exhaustion may result from the CVB3 immune escape mechanism of down-regulating HLA-I antigen presentation. This may ultimately compromise viral clearance by CD8^+^ T cells and favor the reported CVB persistence (*3*, *7*).

In contrast, DRB1*04:01-restricted CVB3 epitopes were consistently immunogenic: They activated a considerable fraction of memory CD4^+^ T cells in most donors, including a sizable T_FH_ subset. This activation was broadly polarized toward T_H_1, T_H_1/17, and T_H_2, but not T_H_17, responses. All these features were similar to those observed for the prototypic viral recall epitope Flu HA_306–318_, suggesting that protective CD4^+^ T cell responses are efficiently mounted and maintained after CVB infection. Beyond supporting cytotoxic responses, CD4^+^ T cells orchestrate broader antiviral defenses (*45*), inducing humoral responses and cross-talking with innate-like lymphocytes. While the T_H_1 arm supports IFN-γ–mediated APC maturation and CD8^+^ T cell cytotoxicity, T_FH_ and T_H_2 responses promote B cell germinal center and Ab maturation (*45*). Hybrid T_H_1/17 differentiation may instead be a double-edged sword (*46*): While it can aid mucosal viral clearance via IL-17–mediated innate cell recruitment, increased IL-17 with T_H_1-skewed inflammation has also been linked to immunopathology, including in CVB3 infection (e.g., myocarditis) (*47*, *48*). Tight regulation of T_H_1/17 skewing may thus be needed to secure viral immunity while minimizing collateral inflammation. Together, these results indicate that antiviral CVB control may predominantly rely on the CD4^+^ T cell arm, compensating the limited cytotoxic CD8^+^ T cell responses. Accordingly, a curated compilation from the Immune Epitope Database (IEDB) (*49*) shows that, in Enteroviruses, the few HLA-I–restricted epitopes identified map mainly to nonstructural proteins and elicit low CD8^+^ T cell response rates, whereas HLA-II–restricted epitopes are more numerous, map largely to structural proteins, and elicit higher CD4^+^ T cell response rates. This is consistent with previous reports of CD4^+^ T cell responses focused on structural proteins also for CVB4 (*50*).

Triggering a CD8-deficient T cell immunity may thus be a general strategy mounted by Enteroviruses to avoid cytotoxic clearance of infected cells. While this limited CD8^+^ T cell immune pressure may favor low-grade viral persistence, the efficient CD4^+^ T cell responses provide therapeutic opportunities to harness them toward protective antiviral immunity. On one side, this may limit CD8^+^ T cell exhaustion, while on the other, it may amplify a productive cross-talk with B cells and innate-like lymphocytes. CD4^+^ T cells can coordinate with mucosal-associated invariant T cells (*51*, *52*) and invariant natural killer T cells (*53*) to stimulate germinal centers. In parallel, innate lymphoid cells (ILCs, particularly ILC3 and ILC2) shape the cytokine milieu that instructs T_H_ polarization and mucosal B cell class switching, reinforcing a feed-forward antiviral loop (*54*). Against this background, a mucosal CVB vaccine may boost more robust CD4^+^ T cell responses, notably T_FH_, promoting immunoglobulin A (IgA) secretion at the gut entry site while recruiting innate-like helpers to accelerate and stabilize protective immunity and compensate the escape from CD8^+^ T cells. Evidence from other viruses shows that mucosal vaccination elicits tissue-restricted immunity that systemic vaccines often miss. For instance, intranasal live attenuated influenza vaccines induce robust nasal IgA and CD4^+^ T cell responses (*55*). Expanding CVB antigen coverage to nonstructural proteins may also overcome the HLA-I “visibility” bottleneck at the gut entry site. As CVB vaccination strategies move into clinical trials (*13*), our findings may inform the design of next-generation candidates, e.g., live attenuated oral CVB vaccines boosting mucosal immunity, as successfully done with the novel Oral Polio Vaccine type 2 against the CVB-related poliovirus (*56*, *57*). These vaccination strategies may be complemented by antiviral therapies (*10*), e.g., the capsid-binding compound pleconaril and enteroviral 2C protein inhibitor fluoxetine (*58*) that may reduce CVB-mediated suppression of IFN and HLA-I pathways. Our validated HLA-I and HLA-II epitope panels also provide immune monitoring tools to track the impact of these interventions on CD8^+^ and CD4^+^ T cells.

Limitations of this study include the use of reductionist in vitro models and high viral loads that may not fully capture the in vivo setting. However, our findings are in line with mouse studies of in vivo CVB3 infection documenting poor CD8^+^ and robust CD4^+^ T cell responses (*59*). Second, we do not provide formal proof that THP-1 macrophages exposed to infected β cells are not, in turn, infected by CVB3. Mitigating this potential, macrophages are reportedly resistant to CVB infection (due to low expression of the CVB entry Coxsackievirus and Adenovirus Receptor and efficient type I IFN responses leading to abortive replication) (*60*). Moreover, the observed peptide display was focused on structural rather than nonstructural CVB3 regions, consistent with a predominant antigen uptake mechanism. Third, low CD4^+^ T cell frequencies only allowed us to infer effector function from phenotypic markers. Fourth, our T cell analyses were limited to healthy donors, preventing causal inference for T1D, i.e., whether these responses are impaired in individuals later developing disease. Addressing this gap will require longitudinal tracking of CVB-reactive CD4^+^/CD8^+^ T cell trajectories during and after natural infection.

In conclusion, CVB3-driven immune escape mechanisms at the intestinal entry site impair antiviral CD8^+^ T cell immunity by stalling precursors on naïve and exhausted effector/memory differentiation states. In contrast, CVB3-reactive CD4^+^ T cells differentiate into polyfunctional memory helper and T_FH_ subsets. These results lend rationale to mucosal vaccination strategies to boost anti-CVB immunity more efficiently and provide tracking tools to follow T cell modifications following natural infection or vaccination.

## MATERIALS AND METHODS

### Experimental design

The objective of this study was to understand the dynamics driving antiviral CVB immunity at the intestinal entry site. Initial proteome analysis of CVB3-infected enterocytes revealed several immune escape mechanisms, including dysregulation of antigen processing and presentation pathways. Following on this observation, we explored the surface expression of HLA-I and HLA-II upon CVB3 infection and downstream modulation of CD4^+^ and CD8^+^ T cell responses. These functional experiments highlighted escape mechanisms from CD8^+^ T cells. In contrast, in vitro CD4^+^ T cell activation was enhanced by HLA-II up-regulation. These findings set the stage for our hypothesis that CVB3 infection in the primary replication site of enterocytes drives limited peripheral CD8^+^ T cell immunity but more robust CD4^+^ T cell responses. To track these CVB3-reactive CD4^+^ and CD8^+^ T cell responses, we identified HLA-I– and HLA-II–bound peptides from two in vitro models of CVB3 antigen presentation: replicative enterocyte infection and macrophage uptake of viral debris. Despite limited viral antigen presentation, we established an HLA-A2– and HLA-DRB1*04:01–restricted candidate epitope panel comprising nonstructural CVB3 peptides, overrepresented in our replicative enterocyte model; and structural CVB3 peptides, enriched in our macrophage uptake model. This panel allowed us to track the frequency and phenotype of circulating CVB3-reactive CD8^+^ T cells, using ex vivo HLA-A2 MMr assays; and CD4^+^ T cells, using AIM assays upon in vitro peptide stimulation. This analysis validated our hypothesis: CVB3-reactive CD8^+^ T cells were developmentally stalled in naïve and exhausted effector/memory states, whereas CD4^+^ T cells comprised a broad range of T_FH_, T_H_1, T_H_2, and T_H_1/17 phenotypes. All in vitro experiments were performed at least in triplicates on at least two separate occasions. Blood samples were processed in batch, and no outliers were excluded; only undersampled data points were excluded if necessary.

### Preparation and titration of CVB3 stocks

Viral stocks were prepared as previously described (*9*). Briefly, CVB3 (Nancy strain; data S1) was propagated in HeLa cells. After overnight infection, supernatants and cells were harvested, subjected to 3 freeze-thawing cycles, and centrifuged at 4000 rpm. Virus particles were then enriched by two rounds of ultracentrifugation to generate a virus stock. The stock was verified by next-generation sequencing, titrated on HeLa cells, and used for experiments without further passaging.

CVB3-GFP, originally obtained as a plasmid construct in which the enhanced GFP gene was cloned into a previously engineered SfiI site immediately downstream of the CVB3 polyprotein start codon within the pMKS1 backbone (*61*), was generated by transfecting HeLa cells. The resulting supernatant was used to produce a new virus stock in HeLa cells. Briefly, HeLa cells were exposed to the supernatant, cultured overnight, and subsequently harvested together with the supernatant. Samples were subjected to 3 freeze-thawing cycles, centrifuged at 4000 rpm, titrated on HeLa cells, and used without further passaging.

### In vitro infection of CaCo2 enterocytes

The first model of CVB3 antigen presentation used the human adherent enterocyte cell line CaCo2 (RRID: CVCL_0025), isolated from a colorectal adenocarcinoma and expressing characteristics of enterocytic differentiation. CaCo2 cells were cultured in Advanced DMEM/F-12 (Thermo Fisher Scientific, #12634028) supplemented with 2% fatty-acid–free bovine serum albumin (BSA; fraction V, Roche, #10775835001), 1% GlutaMAX (Thermo Fisher Scientific, #35050061), 0.02 nM sodium selenite (Sigma-Aldrich, #S9133), 10 mM nicotinamide (Millipore, #481907), 50 μM 2-mercaptoethanol (Sigma-Aldrich, #M6250) and 1% penicillin/streptomycin (Thermo Fisher Scientific, #15140122). Cells with early passage numbers (25 to 29) were seeded in 150-cm^2^ tissue culture flasks (TPP, #90151) at 3.2 × 10^6^ cells per flask and cultured for 3 days or until 60 to 70% confluent. After gentle rinsing with Dulbecco’s phosphate-buffered saline (DPBS), CaCo2 cells were infected with CVB3 (300 MOI) for 1 hour in complete medium without BSA, followed by transfer into fresh prewarmed BSA-free medium. After continuing culture for 10 hours, cells were harvested by gentle scraping in ice-cold dissociation buffer (DPBS and 5 mM EDTA). Cell pellets were washed twice in DPBS and split for flow cytometry analysis or freezing at −80°C as dry pellets for downstream proteomics and immunopeptidomics. For proteomics, nine replicates (5 × 10^6^ cells per each) were generated: four CVB infected and five mock infected. For immunopeptidomics, six replicates (3 × 10^8^ CVB3-infected CaCo2 cells per each) were processed.

Flow cytometry analysis of CaCo2 cells was performed on a BD LSRFortessa II cell analyzer. Surface staining was performed using the monoclonal Ab (mAb) W6/32 to HLA-A/B/C/E (RRID: AB_2917755) and Live/Dead Violet viability marker (Thermo Fisher Scientific, #L34955). Intracellular staining was performed with primary mAbs to VP1 (clone 3A6) (*62*) and dsRNA (RRID: AB_2651015), secondary Abs mouse anti-rat IgG (RRID: AB_465229) and Alexa Fluor 594 (AF594)–conjugated goat anti-mouse IgG (RRID: AB_2762825), and the eBioscience FoxP3 Fix/Perm kit (Thermo Fisher Scientific, #5523).

Reverse transcription quantitative polymerase chain reaction (RT-qPCR) for *HLA-A*, *HLA-B*, *HLA-C*, and HLA-DRB1*04 genes was performed on CVB3- and mock-infected CaCo2 cells, as previously described (*63*). Glyceraldehyde-3-phosphate dehydrogenase was used as the housekeeping gene, and relative quantification was calculated using the 2^−ΔΔ*C*t^ method.

To assess surface CD107a expression, CaCo2 enterocytes were seeded at 30,000 cells per well in 96-well flat-bottom plates and cultured for 12 hours before infection with CVB3-GFP (100 MOI; 1 hour) in serum-free Dulbecco’s modified Eagle’s medium (DMEM). ECN90 β cells were treated similarly but seeded in plates coated with 0.25% fibronectin from human plasma and 1% extracellular matrix from Engelbreth-Holm-Swarm murine sarcoma and cultured in Advanced DMEM/F-12 medium supplemented with 2% BSA, 50 μM 2-mercaptoethanol, 10 mM nicotinamide, sodium selenite (1.7 ng/ml; Sigma-Aldrich), penicillin/streptomycin, and 1% GlutaMAX before infection with CVB3-GFP (50 MOI; 1 hour) in serum-free medium and culture for an additional 16 hours. After gentle washing in DPBS, the culture was continued (20 hours for CaCo2 and 16 hours for ECN90), followed by trypsinization, washing, and processing for flow cytometry. Infection was verified as GFP positivity on viable cells stained with Live/Dead Far Red (Thermo Fisher Scientific, #L10120) and surface staining with Brilliant Violet 786 (BV786)–conjugated mAb to CD107a (RRID: AB_2738458). The median fluorescence intensity (MFI) of CD107a between infected versus noninfected cells was compared to evaluate the increase in surface CD107a expression.

### In vitro uptake of infected ECN90 β cells by DR4/THP-1 macrophages

The second model of CVB3 antigen presentation used the macrophage-differentiated DR4/THP-1 cell line exposed to apoptotic, CVB3-infected human ECN90 β cells derived from neonatal pancreas (HLA-A*02:01/03:01, B*40:01/49:01, and C*03:04/07:01) (*64*). Human THP-1 monocytic cells, derived from a case of acute monocytic leukemia (RRID: CVCL_0006; HLA-A*02:01, B*15:11, C*03:03, DRB1*01:01/15:01, DRA*01:01, DQB1*05:01/06:02, DQA1*01:01/01:02, DPB1*02:01/04:02, and DPA1*01:03/02:02), were lentivirally transduced to stably express HLA-DRB1*04:01 and cultured in RPMI 1640 (Thermo Fisher Scientific, #1870036) supplemented with 20% heat-inactivated fetal bovine serum (FBS; PAN-Biotech, #P30-3306) and 1% penicillin/streptomycin. For differentiation, cells were seeded at 0.5 × 10^6^ cells/ml in 150-cm^2^ flasks and treated with phorbol 12-myristate 13-acetate (PMA; 100 ng/ml; Sigma-Aldrich) for 48 hours (*65*). Following medium removal, cells were gently rinsed once with prewarmed DPBS, and fresh PMA/FBS-free medium was added for an additional 24-hour rest period to allow recovery and stabilization of the adherent macrophage-like phenotype (*66*), followed by a 24-hour coculture with apoptotic CVB3-infected ECN90 β cells (RRID: CVCL_VJ07) at a 1:1 THP-1/ECN90 ratio. ECN90 β cells were driven to apoptosis by CVB3 infection (10 MOI; 72 hours) (*9*). Pulsed DR4/THP-1 cells were harvested by gentle scraping in ice-cold dissociation buffer, washed twice in DPBS, and frozen at −80°C as dry pellets for downstream immunopeptidomics. A total of four replicates (15 × 10^6^ cells per each) was processed.

### Proteomics

Frozen cell pellets for proteomics and immunopeptidomics experiments were thawed in lysis buffer (0.5 ml/10^8^ cells) for 30 min on ice. The buffer consisted of 0.5% IGEPAL CA-630 (Sigma-Aldrich, #I8894), 150 mM NaCl, 50 mM tris-HCl (pH 8.0), and protease inhibitor cocktail (Roche, #11836170001). Lysates were centrifuged at 500*g* for 10 min at 4°C to remove nuclei, followed by 20,000*g* for 45 min at 4°C to pellet residual cell debris.

Lysates were quantified using the Pierce BCA Protein Assay (Thermo Fisher Scientific, #23225) and adjusted to 1 μg/μl in 5% SDS. For each sample, 20 μg of protein was reduced with 10 mM dithiothreitol for 15 min, alkylated with 55 mM iodoacetamide for 15 min, and then digested using the S-Trap micro spin column protocol (ProtiFi). Briefly, proteins were acidified to 2.5% (v/v) phosphoric acid, applied to the S-Trap column, washed with 50 mM triethylammonium bicarbonate in 90% (v/v) methanol, and digested on-column with 2 μg of sequencing-grade trypsin (Promega, #V5111) at 47°C for 1 hour. After washing, peptides were eluted with 50% (v/v) acetonitrile (ACN), 0.1% (v/v) formic acid, vacuum dried, and resuspended in 20 μl of 0.1% (v/v) trifluoroacetic acid (TFA) and 1% (v/v) ACN.

Peptides were separated on an Ultimate 3000 RSLCnano system (Thermo Fisher Scientific) using a PepMap C18 EASY-Spray column (75 μm × 50 cm, 5 μm) in 0.1% formic acid and coupled to a Q Exactive HF-X mass spectrometer (Thermo Fisher Scientific). A 2 to 35% ACN gradient in water containing 1% DMSO and 0.1% formic acid was run at 250 nl/min. Ionization was via EASY-Spray at 2000 V with a transfer tube temperature of 250°C. Data were acquired in proteomics data-dependent acquisition (DDA) mode: a full MS1 scan [mass/charge ratio (*m/z*): 320 to 1600; resolution: 60,000; automatic gain control: 3 × 10^6^, maximum injection time: 45 ms] followed by 12 tandem MS (MS/MS) scans (resolution: 30,000; automatic gain control: 5 × 10^5^, maximum injection time: 54 ms) using a 1.3 *m/z* isolation width, +2 to +4 charge states, normalized higher collisional dissociation energy: 28%, and 30-s dynamic exclusion. All data were recorded in profile mode.

Raw MS data were processed with FragPipe v22.0 (LFQ-MBR workflow) using the same database as for immunopeptidomics (see below). Searches were performed with trypsin specificity (up to two missed cleavages), a precursor mass tolerance of ±20 parts per million (ppm), and a fragment mass tolerance of ±20 ppm. Carbamidomethylation of cysteine (+57.02146 Da) was set as a fixed modification, while methionine oxidation and N-terminal acetylation were set as variable modifications. Peptide-spectrum matches were filtered to 1% peptide spectrum match (PSM)-level false discovery rate (FDR) with Percolator, and protein inference was performed with ProteinProphet at 1% protein-level FDR. Label-free quantification was conducted with IonQuant (*67*) (MaxLFQ enabled, match-between-runs active, *m/z* tolerance: 10 ppm, retention time (RT) tolerance: 0.4 min) with normalization across runs. MSBooster (*68*) was also applied.

Label-free quantification (MaxLFQ intensity) data were analyzed in FragPipe-Analyst (*69*). Proteins with ≥70% nonmissing values were retained, and missing values were imputed using the Perseus-type method. Differential expression was assessed with Limma, applying a Benjamini-Hochberg–adjusted *P* ≤ 0.05. Functional enrichment (Gene Ontology Biological Process, Kyoto Encyclopedia of Genes and Genomes, and Reactome) guided manual selection of proteins related to enterocyte differentiation, antigen processing and presentation, IFN signaling and autophagy.

### Functional T cell priming assays

To assess the effect of HLA-II up-regulation on antigen presentation, CaCo2 cells were infected with CVB3 (10 MOI; 18 hours), followed by pulsing with the HLA-DR4–restricted Flu HA_306–318_ peptide (PRYVKQNTLKLAT; 10 μM) or DMSO diluent for 1 hour. CaCo2 cells were then cocultured at a 1:1 ratio with a Flu HA–reactive CD4^+^ T cell clone for 3 hours, after which brefeldin A (BFA; 5 μg/ml; BioLegend, #420601) was added for an additional 1 hour. Cells were stained with Live/Dead Far Red, followed by intracellular staining with fluorescein isothiocyanate (FITC)–conjugated TNF-α mAb (RRID: AB_315258). TNF-α expression in CD4^+^ T cells was measured by MFI as a readout of T cell activation.

To assess the effect of HLA-I down-regulation on antigen presentation by CVB3-infected CaCo2 cells, CD8^+^ T cell–mediated killing was assessed in real time using the Incucyte S3 Live-Cell Analysis System (Sartorius). A stable CaCo2 cell line expressing the NucLight Red nuclear label (mKate2; Sartorius, #4717) was generated by lentiviral transduction following the manufacturer’s instructions. Cells (20,000 per well, 96-well flat-bottom plates; TPP, #92096) were infected with CVB3-GFP at 10 MOI. After 1 hour, unbound virions were removed by washing with prewarmed medium, and fresh medium was added. CVB3_1246–1254_ TCR-transduced primary CD8^+^ T cells (*9*) were then added at a 1:1 effector-to-target ratio. Control conditions included the following: (i) infected CaCo2 cells without CD8^+^ T cells, (ii) infected CaCo2 cells with irrelevant CD8^+^ T cells transduced with the PPI_15–24_ TCR 1E6 (*9*), (iii) infected CaCo2 cells pulsed with CVB3_1246–1254_ peptide (1 μM) and cocultured with CVB3-reactive CD8^+^ T cells, and (iv) mock-infected CaCo2 cells cocultured with CVB3-reactive CD8^+^ T cells. Plates were scanned every 2 hours for 68 hours with phase contrast, green (GFP; acquisition time: 300 ms), and red (mKate2 Red; acquisition time: 400 ms) channels at ×10 magnification. Green and red object masks were generated using surface fit segmentation with a threshold of 2 calibration units. Fold changes in CaCo2 cell numbers were quantified as the red object count per image normalized to the value at 0 hours. Percentages of infected cells were determined by calculating the ratio of green and red area overlap to the total red area.

### Immunopeptidomics

Before IP of pHLA complexes from cell lysates, mAb-coated, cross-linked monoresins were prepared. Protein A Sepharose beads (Abcam, #ab270308) were used to immobilize mouse IgG2a mAbs purified from W6/32 hybridomas (RRID: CVCL_7872) for pan–HLA-I immunopeptidome capture and L243 hybridomas (RRID: CVCL_4533) for HLA-DR capture. Protein G Sepharose beads (Abcam, #ab270309) were used to immobilize mouse IgG1 mAbs purified from IVA12 hybridomas (RRID: CVCL_G223) for pan–HLA-II immunopeptidome capture. Beads were washed to replace the ethanol storage solution with PBS, then mixed with mAbs at a ratio of 100 μl of actual beads (originally supplied as a 50% agarose slurry in 20% ethanol) per 2 mg of mAb, and incubated with gentle rotation for 1 hour at 4°C. mAbs were subsequently cross-linked to the beads using 40 mM dimethyl pimelimidate dihydrochloride (Sigma-Aldrich, #D8388).

IPs were performed according to the CVB3 antigen presentation model used: CaCo2 (W6/32 and IVA12) and DR4/THP-1 cells (L243). Monoresin mixes were prepared by loading a mAb equivalent of 1 mg per up to 10^8^ cells. mAb-coated beads were incubated with cleared lysates for 16 hours at 4°C with gentle rotation. Monoresins were collected by gravity flow and washed sequentially with 50 mM tris buffer in four steps: (i) 150 mM NaCl and 5 mM EDTA, (ii) 150 mM NaCl, (iii) 450 mM NaCl, and (iv) no added salt. Peptides were eluted from the captured pHLA complexes using 10% acetic acid and then passed through a 5-kDa cutoff filter (Millipore, #UFC3LCCNB-HMT), and the flow-through was vacuum dried. Peptides were resuspended in 1% ACN and 0.1% TFA, desalted on Pierce C18 tips (#84850), and dried by SpeedVac. The final eluates were resuspended in 20 μl of 1% ACN with 0.1% formic acid before MS analysis.

Samples were acquired on a nano-UHPLC system (nanoElute, Bruker Daltonics) coupled to a Trapped Ion Mobility Spectrometry–Time of Flight (timsTOF) SCP (Bruker). Peptides were loaded onto an Aurora C18 column (25 cm × 75 μm, 1.7 μm; IonOpticks) with 0.1% formic acid at 800 bar for 9 min at 50°C. Separation was performed at 50°C using a linear gradient of 2 to 20% ACN in 0.1% acetic acid over 60 min and then 20 to 37% ACN in 6 min at 150 nl/min. Electrospray ionization (CaptiveSpray source, Bruker) settings were 180°C, 1400 V, and dry gas (3 l/min). MS was acquired in DDA-parallel accumulation/serial fragmentation (PASEF) mode with one MS survey TIMS-MS and 10 PASEF MS/MS scans per cycle. Ion accumulation and ramp times were 166 ms each, with an ion mobility range of 1/*K*_0_ = 1.7 to 0.7 V·s·cm^−2^ and *m/z* of 100 to 1700. Multiple-charged and single-charged ions with *m/z* ≥ 700 were selected if intensity ≥ 500 arbitrary units (a.u.) and resequenced until a target of 20,000 a.u. was reached. Collision energies for immunopeptidomics were 70 eV at 1/*K*_0_ = 1.7 V·s·cm^−2^; 40 eV at 1/*K*_0_ = 1.34 V·s·cm^−2^; 40 eV at 1/*K*_0_ = 1.1 V·s·cm^−2^; 30 eV at 1/*K*_0_ = 1.06 V·s·cm^−2^; and 20 eV at 1/*K*_0_ = 0.7 V·s·cm^−2^.

timsTOF data were processed in FragPipe v22.0 (HLA nonspecific workflow) using MSFragger (*70*–*72*) against a concatenated target-decoy database comprising the UniProtKB/Swiss-Prot human proteome (reviewed entries, downloaded on 17 January 2024), and a six-frame translation of the genome sequenced from the CVB3 strain was used (data S1; including all ORFs ≥7 amino acids); decoys were generated in Philosopher using the reversed sequence method. Searches were run with nonspecific termini, peptide length of 7 to 25 amino acids, no fixed modifications, and variable modifications including oxidation (M; +15.9949 Da), cysteinylation (C; +119.0041 Da), N-terminal acetylation (+42.0106 Da), pyro-glutamate formation from N-terminal Q (−17.0265 Da) or E (−18.0106 Da), with up to three variable modifications per peptide. Precursor and fragment mass tolerances were ± 20 ppm. PSMs were rescored with MSBooster (*68*) (DIA-NN models for RT and spectra) and validated with Percolator using a 1% FDR threshold at the PSM, ion, and peptide levels; no protein-level FDR filter was applied. Label-free quantification was performed with IonQuant (*67*) (*m/z* tolerance: 10 ppm, RT tolerance: 0.4 min, ion mobility tolerance: 0.05 1/*K*_0_, match-between-runs enabled, MaxLFQ normalization). Motif deconvolution was performed using MHCMotifDecon (*73*), and in silico binding predictions were carried out with NetMHCpan 4.2 (*74*) and NetMHCIIpan 4.3 (*75*). Peptides with a conservative predicted rank ≤ 4% were classified as binders.

### HLA-A*02:01 binding assays

Peptide binding to HLA-A*02:01 was assessed using a T2 stabilization assay. Transporter Associated with Antigen Processing (TAP)-deficient T2 cells (HLA-A02:01^+^) were incubated overnight at 26°C and 5% CO_2_ in serum-free RPMI 1640 with 10 μM 9-mer peptides (>95% purity; Synpeptide), including Flu MP_58–66_ as a positive control and NY-ESO-1_125–133_ as a negative control. Cells were washed and resuspended in prewarmed RPMI 1640 containing BFA (5 μg/ml) to block new HLA-A2 export. Samples were collected every hour for 5 hours, stained with HLA-A2 mAb (RRID: AB_1659245) and Live/Dead Far Red, and analyzed on a BD LSRFortessa II flow cytometer. MFI values were normalized to t0 and fitted to a one-phase exponential decay model using GraphPad Prism 8 to calculate half-lives.

For fold-change determination, T2 cells were pulsed overnight at 26°C with peptides at 0.5, 5, or 50 μM in serum-free medium, shifted to 37°C, and treated with BFA (5 μg/ml) for 4 hours before HLA-A2 staining as above. DMSO-treated cells served as the baseline. Peptides were classified as functional binders if they induced ≥2.3-fold HLA-A2 MFI over the DMSO baseline.

### HLA-DRB1*04:01 binding assays

Peptides predicted to bind HLA-DRB1*04:01 (DR4) (>95% purity; Synpeptide) were assessed using a competitive inhibition assay. DR4/THP-1 cells were pulsed in AIM-V serum-free medium (Thermo Fisher Scientific, #12055083) with serial dilutions of candidate peptides (0.000001 to 400 μM) for 2 hours at 37°C, followed by addition of the DR4-restricted Flu HA_306–318_ peptide at 0.025 μM for an additional 1 hour. This concentration of Flu HA was selected to induce ∼25% of the maximal TNF-α response. After washing, cells were cocultured in AIM-V medium with a DR4-restricted Flu HA_306–318_–reactive CD4^+^ T cell clone at a 1:5 T cell/THP-1 ratio. BFA (5 μg/ml) was added after 3 hours, and cocultures was maintained for a total of 6 hours. Cells were then stained with Live/Dead Far Red, fixed/permeabilized, and intracellularly stained with FITC-conjugated anti–TNF-α. Samples were analyzed on a BD LSRFortessa II flow cytometer. The percent maximal TNF-α response was plotted against competitor peptide concentration, and curves were fitted in GraphPad Prism 8 using a nonlinear (three parameters) with least squares fitting, constraining bottom = 0 and median inhibitory concentration (IC_50_) > 0. Peptides were classified as DR4 binders if they exhibited an IC_50_ < 400 μM.

### Human duodenal organoids

Primary hDOs (HLA-A*01:01/02:01, B*08:01/15:01, and C*03:04/07:01) were established from deidentified surgical specimens under the Tampere University Hospital ethics approval (ETL R18082). Intestinal crypts containing stem cells were isolated (2 mM EDTA for 30 min at 4°C) (*76*), seeded in 50% Matrigel, passaged, and maintained as three-dimensional (3D) cultures in expansion medium (*77*). Organoids were mechanically disrupted, plated as 2D monolayers on diluted Matrigel, and differentiated 24 hours before infection with supplemented Advanced DMEM/F-12 medium (*77*) containing EGF (50 ng/ml; PeproTech, #AF-100-15), LDN-193189 (0.1 μM; Selleckchem, #S2618), and R-Spondin-1 (250 ng/ml; Peprotech, #120-38). Monolayers (∼10^7^ cells) were infected with CVB3 (MOI 100) for 15 hours. Parallel wells were fixed in 4% paraformaldehyde and stained for VP1 (clone 3A6), caspase-3/7 fluorogenic substrate (Sigma-Aldrich, #SCT105), and Hoechst 33342 to verify infection and apoptosis, respectively. HLA-I complexes were immunoprecipitated with W6/32 mAb; peptides were eluted, acquired on a timsTOF SCP mass spectrometer, and analyzed as above.

### Study participants, HLA typing, and CVB serology

Study participants (table S2) were recruited in Paris after providing written informed consent, under ethics approval 21.01064.000002 (Ouest IV, Nantes). HLA typing was performed by DKMS (Tübingen, Germany), and donors positive for HLA-A*02:01, HLA-DRB1*04:01, or both were selected for T cell assays. Peripheral blood was collected in 9-ml sodium heparin tubes and PBMCs isolated and cryopreserved following our established protocols (*30*, *78*). All experiments were performed on frozen/thawed PBMC samples. Neutralizing Ab titers against the six CVB serotypes were measured with a plaque neutralization assay as described (*79*).

### Combinatorial HLA-I MMr assays

Fluorescent-labeled, peptide-loaded HLA-A2 MMrs were generated as previously described (*9*, *29*–*32*), conjugated to fluorochrome-labeled streptavidins at a 1:4 molar ratio, and used at final concentrations of 8 to 27 nM, adjusted to normalize for the variable staining index of each streptavidin to ensure clear visualization of distinct double-MMr^+^ populations for each fluorochrome pair. PBMCs were thawed in prewarmed AIM-V medium and rested for 1 hour at 37°C with 50 nM dasatinib, followed by negative magnetic enrichment of CD8^+^ T cells (RRID: AB_2728716). Staining with combinatorial, double-coded MMr panels was performed for 30 min at room temperature (RT) in DPBS containing dasatinib, after which phenotyping mAbs were added for 30 min at 4°C: CD8-BUV496 (RRID: AB_2916884), CD45RA-BUV737 (RRID: AB_2870168), PD-1–BB700 (RRID: AB_2744348), CD39-BV605 (RRID: AB_2750430), CCR7-PE/Cy7 (RRID: AB_11126145), CD95-APC/Fire750 (RRID: AB_2629736), TCF-1–AF488 (RRID: AB_2916388), and Live/Dead Aqua (Thermo Fisher Scientific, #L34957). After washing, cells were acquired on a Cytek Aurora spectral flow cytometer and analyzed using FlowJo v10 and GraphPad Prism 10. Positive control peptides included MelanA_26–35_ ELA (naïve self-control; ELAGIGILTV; IEDB #12941) and Flu MP_58–66_ (recall viral control; GILGFVFTL; IEDB #20354). All MMr^+^ cells were pooled into a concatenated file for t-SNE analysis in FlowJo using all phenotyping panel markers (except the fluorochrome channels assigned to MMrs, CD8, and viability); iterations were set to 1000, perplexity to 30, and learning rate (η) to 2482. The k-nearest neighbors algorithm was set to Exact (Vantage Point Tree), the gradient algorithm to Barnes-Hut, and learning configuration to auto (opt-SNE). Dimensionality reduction was complemented with PhenoGraph clustering (*k* = 30), and results were exported for t-SNE map replotting in Python.

### AIM assays

PBMCs were thawed and rested for 3 hours at 37°C and 5% CO_2_ in AIM-V medium before stimulation. A total of 3 to 4 × 10^6^ PBMCs was plated in 1 ml of AIM-V medium per well in 48-well plates, supplemented with a blocking anti-CD40 mAb (RRID: AB_10839704; 1 μg/ml) to limit CD40L down-regulation. Cells were stimulated for 24 hours with individual DRB1*04:01-restricted CVB3 peptides (10 μM), DMSO diluent as a negative control, or Flu HA_306–318_ as positive effector/memory control.

Following stimulation, staining was performed in three sequential steps separated by DPBS washes in between. First, cells were incubated at RT for 30 min with chemokine receptor mAbs: CCR4-BV421 (RRID: AB_2737663), CCR6-BUV737 (RRID: AB_2870109), CXCR3-RB705 (RRID: AB_3685840), and CXCR5-BV480 (RRID: AB_2739586). Second, Live/Dead Green viability staining was performed at RT for 20 min (Thermo Fisher Scientific, #L23101). Third, cells were incubated at 4°C for 30 min with the remaining mAbs to explore T cell phenotype and activation: CD3-BUV496 (RRID: AB_2870222), CD4-BV805 (RRID: AB_2870176), CD8-BUV563 (RRID: AB_2870199), HLA-DR–BUV661 (RRID: AB_2870252), CD25-BV711 (RRID: AB_2738037), CD127-BV785 (RRID: AB_11219610), CD45RA-BV605 (RRID: AB_11126164), CD69-PE/Dazzle594 (RRID: AB_2564277), CD134 (OX40)–PE/Cy5 (RRID: AB_2894615), CD137 (4-1BB)–PE/Cy7 (RRID: AB_2207741), CD154 (CD40L)–PE (RRID: AB_2751142), and PD-L1–PE/Fire810 (RRID: AB_2894668). Samples were acquired on a Cytek Aurora spectral flow cytometer and analyzed with FlowJo v10 and GraphPad Prism 10. Boolean gating identified all AIM combinations per donor; combinations absent in matched DMSO stimulation controls were, after redundancy reduction by OR gating, defined as antigen specific and used for downstream analyses.

### Statistical analysis

Data are shown as median (range) or mean ± SD. Significance was assessed using two-tailed tests with a cutoff value of α = 0.05, as detailed for each figure.
